# Insights and implications of sexual dimorphism in osteoporosis

**DOI:** 10.1038/s41413-023-00306-4

**Published:** 2024-02-18

**Authors:** Yuan-Yuan Zhang, Na Xie, Xiao-Dong Sun, Edouard C. Nice, Yih-Cherng Liou, Canhua Huang, Huili Zhu, Zhisen Shen

**Affiliations:** 1 Key Laboratory of Drug-Targeting and Drug Delivery System of the Education Ministry and Sichuan Province, Sichuan Research Center for Drug Precision Industrial Technology, West China School of Pharmacy, Sichuan University, Chengdu, 610041 China; 2 West China School of Basic Medical Sciences & Forensic Medicine, Sichuan University, Chengdu, 610041 China; 3 Department of Biochemistry and Molecular Biology, Monash University, Clayton, VIC 3800 Australia; 4 Department of Biological Sciences, Faculty of Science, National University of Singapore, Singapore, 117543 Republic of Singapore; 5 Department of Biotherapy, Cancer Center and State Key Laboratory of Biotherapy, West China Hospital, and West China School of Basic Medical Sciences & Forensic Medicine, Sichuan University, Chengdu, 610041 China; 6 Key Laboratory of Birth Defects and Related Diseases of Women and Children of Ministry of Education, Department of Reproductive Medicine, West China Second University Hospital of Sichuan University, Chengdu, China; 7 Department of Otorhinolaryngology and Head and Neck Surgery, The Affiliated Lihuili Hospital, Ningbo University, 315040 Ningbo, Zhejiang China

**Keywords:** Osteoporosis, Osteoporosis

## Abstract

Osteoporosis, a metabolic bone disease characterized by low bone mineral density and deterioration of bone microarchitecture, has led to a high risk of fatal osteoporotic fractures worldwide. Accumulating evidence has revealed that sexual dimorphism is a notable feature of osteoporosis, with sex-specific differences in epidemiology and pathogenesis. Specifically, females are more susceptible than males to osteoporosis, while males are more prone to disability or death from the disease. To date, sex chromosome abnormalities and steroid hormones have been proven to contribute greatly to sexual dimorphism in osteoporosis by regulating the functions of bone cells. Understanding the sex-specific differences in osteoporosis and its related complications is essential for improving treatment strategies tailored to women and men. This literature review focuses on the mechanisms underlying sexual dimorphism in osteoporosis, mainly in a population of aging patients, chronic glucocorticoid administration, and diabetes. Moreover, we highlight the implications of sexual dimorphism for developing therapeutics and preventive strategies and screening approaches tailored to women and men. Additionally, the challenges in translating bench research to bedside treatments and future directions to overcome these obstacles will be discussed.

## Introduction

Osteoporosis, the most prevalent metabolic bone disease, affects approximately 200 million individuals worldwide and is characterized by a decrease in bone mineral density (BMD) and progressive microarchitecture deterioration of bone tissue. These changes are associated with an increased risk of fragility fractures.^1^ Prior to a fracture, osteoporosis is usually asymptomatic, but when a fracture occurs, it can be extremely painful and even life-threatening.^2^ Fractures in the spine and hip, in particular, can lead to mortality, with approximately 20% of individuals experiencing osteoporotic hip fractures dying within six months.^3^ As life expectancy increases and the baby boomer generation ages, the burden of osteoporosis and fractures is expected to grow rapidly, resulting in a significant impact on morbidity and mortality rates.^4,5^ Therefore, osteoporotic fractures are considered one of the most significant public health priorities by the World Health Organization.^6^

Osteoporosis and its complications exhibit particularly pronounced sex-related differences.^7^ Men are less susceptible than women to developing primary osteoporosis and osteoporotic fractures due to their larger, stronger bones and slower rate of bone loss in adulthood.^8,9^ However, men are more prone to secondary osteoporosis and have a higher overall mortality rate from osteoporotic fractures.^10^ These sexual dimorphisms in the prevalence and prognosis of osteoporosis and associated complications may be attributed to sex-specific pathological mechanisms, including estrogen receptors (ERs) and associated pathways, which are key regulators of bone homeostasis.^1,11–13^ Despite this, extensive preclinical research and clinical studies have been conducted in both male animal models and men, primarily driven by concern about the influence of the hormonal cycle (e.g., menstrual cycle) on outcomes as well as the classification of women as “protected subjects” in clinical trials.^14,15^

Currently, the sex of the patient, an important factor in the prevention, screening, and treatment of osteoporosis and fractures, has not been well taken into account. Most medications, guidelines, screening, and fracture risk prediction methods have been developed for women and subsequently adapted for use in men.^16^ Therefore, gaining further insights into the sex differences in osteoporosis, especially the underlying pathological mechanisms, can help guide the development of age- and sex-specific preventive, screening, and therapeutic strategies for osteoporosis and its complications.

A better understanding of the sex-specific pathophysiology of osteoporosis can contribute to the development of novel, tailored therapeutics and preventive approaches. This review aims to address the sexual dimorphism of osteoporosis, with a focus on the pathogenic mechanisms related to sex chromosome abnormalities, steroid hormones, and psychological stress in osteoporosis. Despite the intriguing and exciting research in this field, numerous issues remain to be further addressed to effectively translate transformative discoveries into beneficial treatments. Therefore, in this review, we also discuss the challenges and potential solutions that can accelerate the development of tailored therapeutics and screening strategies for osteoporosis.

## Disparities in osteoporosis epidemiology according to sex

Aging, a decline in sex steroids, long-term use of glucocorticoids and diabetes contribute to osteoporosis by altering the balance between bone formation and resorption.^17^ Osteoporosis is commonly associated with postmenopausal women, which can be attributed to their longer lifespans and the sharp decline in estrogen levels that they experience.^18^ In addition to primary osteoporosis, which typically occurs due to aging and/or hormonal factors, secondary osteoporosis is associated with pharmacological agents (such as glucocorticoids (GCs)) or underlying diseases such as diabetes.^19–21^ Notably, secondary osteoporosis is more prevalent in men than in women. Additionally, men face a higher risk of mortality following osteoporotic fractures than women. Despite limited evidence, there is still controversy surrounding sex-specific differences in GC-induced and diabetic osteoporosis.

### Primary osteoporosis in older women and men

Osteoporosis is a metabolic disease that reduces bone mass and impairs bone microarchitecture, leading to skeletal fragility and an elevated risk of fractures.^22^ Historically, osteoporosis has been primarily associated with postmenopausal women, considering it a bone health issue that predominantly affects this group.^23^

**Fig. 1:**
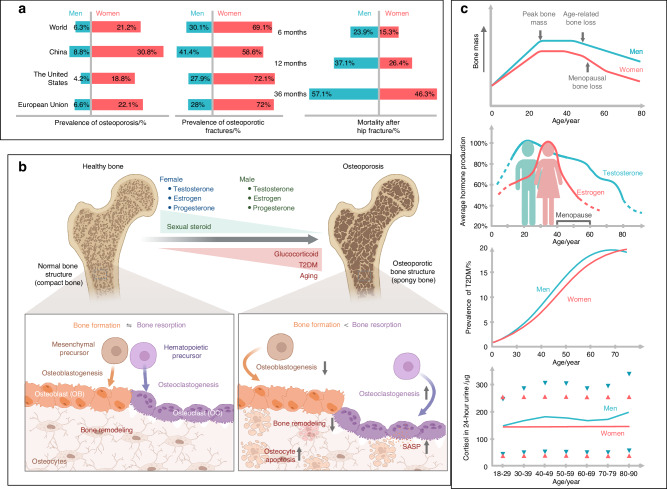
The prevalence of osteoporosis in men and women throughout their lifetimes. **a** Left: The prevalence of osteoporosis in women and men aged 50 and older in selected countries. The sex-specific prevalence rates of osteoporosis in the world, China, European Union (EU) countries, and the United States (US) are presented for women and men aged 50 years and older. The criterion of the World Health Organization was applied for the diagnosis of osteoporosis. The following data sources were used to determine osteoporosis prevalence: osteoporosis prevalence worldwide and in EU countries (https://www.osteoporosis.foundation/facts-statistics/epidemiology-of-osteoporosis-and-fragility-fractures); osteoporosis prevalence in China, the survey of the China Ministry of Health; and osteoporosis prevalence in the US, The Centers for Disease Control and Prevention. Middle: The sex-specific prevalence of osteoporotic fractures in selected countries. Unlike osteoporosis, the population-based prevalence of osteoporotic fractures is difficult to obtain for men and women by country due to the lack of standard diagnostic criteria. The data represent the proportions of hip fractures in men and women worldwide, China, EU countries, and the US. Data on hip fractures in China were adopted from a study in Hefei, China.^526^ Data for hip fractures worldwide, in EU countries, and in the US originated from the following data source: (https://www.osteoporosis.foundation/facts-statistics/epidemiology-of-osteoporosis-and-fragility-fractures). Right: The cumulative mortalities among male hip fracture patients were higher than those of female patients at 6, 12, and 36 months. Data were adopted from the study in Denmark.^36^ **b** The balance of bone formation and bone resorption changes during a lifetime due to decreased sex steroids, increased glucocorticoids, T2DM, and aging. Bone resorption can outweigh formation because of decreased osteoblastogenesis, enhanced osteoclastogenesis, reduced bone remodeling, and cell senescence. **c** Bone mass (upper), average sex hormone production (upper middle), prevalence of T2DM (lower middle), and urine cortisol levels (below) in men and women throughout their lifetime. The dots representing 24 h urine cortisol levels, shown as the 2.5th and 97.5th percentiles, were adapted from a study in Switzerland.^48^ SASP senescence-associated secretory phenotype

In fact, women over the age of 50 have an osteoporosis prevalence approximately four times higher than men in the same age group worldwide, making osteoporosis the most common cause of fractures among the elderly^24,25^ (Fig. 1a). Although population-based prevalence data for osteoporotic fractures are lacking, current data demonstrate sex disparities in hip fractures^26^ (Fig. 1a). In the European Union (EU), only 21% of the 20 million people with osteoporosis are male; however, it is important to note that men have a higher mortality rate following an osteoporotic fracture than women.^10^

Fracture patterns resulting from osteoporosis differ between males and females. Hip fractures, the most severe complication of osteoporosis, occur at a lower incidence in men than in women, with approximately one-third of cases occurring in males.^27^ In a large-scale study of fractures conducted in the United States (US), male populations accounted for 27.9% of hip fractures in 2010, and this proportion is projected to increase to 37.8% by 2030.^28^ In the MrOS MsOS (Hong Kong) studies, after a 14-year follow-up, the incident vertebral fracture rate was higher in women than in men, regardless of whether they had baseline osteoporotic vertebral deformity.^29^ According to a nationwide cohort study in South Korea that enrolled 73 717 patients with osteoporotic fractures, the incidence rate of subsequent fractures within 24 months was higher in females than in males (10.37 vs. 9.14 per 100 person-years, respectively).^30^ It has been reported that vertebral bone and long bones are formed by different stem cell populations, making vertebral bone a feasible target for a distinct set of diseases, such as tumor metastases.^31^ Furthermore, the differences in fracture patterns between the sexes may be because skeletal stem cell-mediated bone regeneration depends on estrogen signaling in female mice but not in male mice.^32^

The mortality rate following osteoporotic fractures is greater in men than in women. As reported by a nine-year cohort study of individuals over the age of 60 with osteoporotic hip fractures, women have a predominance of fractures (64.68%), whereas men suffer higher mortality rates (20.42%; 95% CI: 13.76–27.08).^33^ Men had a slightly higher likelihood of dying from all-cause mortality within a year after a hip fracture than women (22.8% vs. 19.5%).^34^ A prospective study revealed that hip fracture decreased life expectancy, with men having a larger reduction than women.^35^ Even after controlling for age, fracture sites, and medications, long-term survival analysis disclosed that the higher mortality for men is overwhelmingly significant compared to women (HR 1.70, 95% CI: 1.65–1.75, *P* < 0.001) (Fig. 1a).^36^

In addition to the considerably high mortality rate associated with hip fractures in men, the mortality risk remains significant following most osteoporotic fractures. According to data from the Korea National Health Insurance Service, the cumulative mortality rate in the first year after initial distal radius fractures was higher in males than in females (2.24% vs. 1.30%).^37^ Altogether, it is important to recognize that osteoporosis not only affects older women but also causes serious health issues for male populations.^38^

### GC-induced osteoporosis

GC-containing medications, including cortisone, prednisone, dexamethasone, and hydrocortisone, are the leading cause of secondary osteoporosis and severe osteoporotic fractures in both sexes.^39^ GCs play a crucial role in maintaining skeletal homeostasis, but insufficient or excessive levels can lead to osteoporosis and fracture. Hip fracture risk is increased by endogenous GC deficiency conditions such as Addison’s disease, whereas long-term usage of synthetic GCs frequently induces rapid bone loss and an enhanced risk of fractures in a time- and dose-dependent way.^40^ The risk of vertebral fracture is nearly three times higher, and the risk of hip fracture is approximately twice as high in individuals taking oral GCs.^41^ Although the degree of GC-induced bone loss varies greatly and no clear predictors have been validated for individual fracture risk, it is recommended to undergo a BMD test or receive osteoporosis treatment within six months of initiating GC therapy.^42^

The sexual differences in susceptibility to and microarchitecture changes caused by GC-induced osteoporosis are contradictory. The prevalence of GC-induced osteoporosis is slightly higher in men than in women, with rates of 67% and 59%, respectively.^43^ However, the prevalence of GC-induced osteoporosis was not significantly different between men and women in a large Japanese study with 25 569 patients. The study included a population with a mean age of 68.5 years, with 90.5% of patients being over the age of 50.^44^

GCs have been shown to reduce areal BMD at the femoral neck in women, as well as the total femur and femoral neck in men.^45^ In postmenopausal women, GC-induced osteoporosis is associated with changes in trabecular and cortical changes, whereas in men, it results in diminished trabecular thickness and connectivity.^46^ Notably, only 12% of men and 23% of women who had not received prior osteoporosis management underwent BMD testing or received osteoporosis treatment while they were undergoing oral GC therapy, indicating that chronic GC users do not adequately undergo osteoporosis prevention treatment, with men being more undertreated than women.^47^

Several factors conceal sexual dimorphism in GC-induced osteoporosis. As reported by a large population-based multicentric study, the 24-h urine excretion of cortisol increases with age in men but not in women (Fig. 1c).^48^ These sex-specific cortisol changes may contribute to age-related osteoporosis, making the elucidation of sexual dimorphism even more difficult. GCs, whether endogenous overproduction or exogenous administration, may induce impaired insulin sensitivity and hyperglycemia, indirectly contributing to bone loss and osteoporosis.^49^ The changes in sex hormones make the situation more complex.^43^ GCs synergistically aggregate the deteriorating effects of estrogen decline in bone loss, resulting in higher rates of fragility fractures in postmenopausal women (41.9%) than in premenopausal women (5.4%).^50^ Stanozolol derived from dihydrotestosterone (DHT) can enhance BMD and improve biomechanical properties in GC-induced osteoporosis, possibly due to the anabolic effects of androgen on the skeletal system.^51,52^ It is challenging to examine the sexual dimorphism of GCs in osteoporosis because they are widely used, from rheumatic ailments in women to renal problems in children. Additionally, the majority of current research on GC-induced osteoporosis focuses on older populations, leaving limited information regarding the impact on premenopausal women and men under the age of 50.^46^

### Diabetic osteoporosis

Growing evidence suggests an elevated risk of osteoporosis and osteoporotic fractures in patients with diabetes.^53–58^ Patients with type-1 diabetes mellitus (T1DM) often have lower bone strength and a higher risk of fractures.^59,60^ Type-2 diabetes mellitus (T2DM) leads to skeletal abnormalities, including increased cortical porosity and a deficit in bone material properties. Patients with T2DM face a higher risk of fractures, despite their BMD being increased or within the normal range.^56–58^ In both Western European and East Asian populations, the prevalence of T2DM is higher in men than in women.^61,62^ The sex differences in T2DM may be due to sexual dimorphism in the utilization of energy and metabolic substrates in metabolic organs, such as adipose tissue, muscle, and the liver.^63,64^ It is plausible that these sexual variations in metabolic organs also contribute to the sex-specific differences in diabetic osteoporosis.

T1DM patients have a sexually dimorphic prevalence of osteoporosis, with women being at higher risk. Both men and women with T1DM have a 2- and 4-fold increased risk of hip and lumbar spine fractures, respectively.^60^ A prospective, multicenter study found that the odds of fractures were higher in prepubertal females (OR 2.81, 95% CI 1.21–6.52) than in postpubertal males (OR 2.44, 95% CI: 1.11–5.38).^65^ In line with T1DM, the risks of osteoporosis in T2DM patients are higher in females than in males.^66,67^ The prevalence of osteoporosis in female T2DM patients was significantly greater than that in male patients (21.9% vs. 13.0%), suggesting a sex difference in T2DM-associated secondary osteoporosis.^59^ Increased vertebral fractures were reported in female T2DM patients (RR 1.3), while neither prevalent nor incident morphometric fractures were increased in male patients, although the point estimate (HR 1.28) was similar.^66^ Similarly, the Dubbo osteoporosis epidemiology study reported greater incident fracture rates in women with T2DM than in men (24.5/1 000 vs. 12.7/1 000 person-years).^68^

The mortality risk following fracture in women and men with T2DM remains controversial. According to a population-based cohort study, men have greater total postfracture death rates than women.^69^ A meta-analysis including 22 cohort studies also revealed a higher mortality risk in men than in women within five years after a hip fracture.^70^ However, the Dubbo study reported that after adjusting for confounders, the risk of mortality in female T2DM patients was higher than that in males (HR 1.68, 95% CI 1.30–2.16 vs. HR 1.16, 95% CI: 0.91–1.49). Given that the participants in the Dubbo study had average ages that were higher than 65, it is important to note that the average ages should be further evaluated to investigate the sex difference in mortality risk.^68^

Analyzing sex dimorphism in diabetic osteoporosis poses significant challenges. One major challenge is that T1DM starts at a much earlier age than T2DM, even during adolescence. The age differences, coupled with variations in the status of sex hormones among patients participating in clinical observations of T1DM and T2DM, complicate the analysis. Additionally, T2DM patients have many comorbidities, such as rheumatic arthritis.^71^ Hence, these patients require treatment with GCs, which further worsens the osteopathy of these patients and adds complexity to studying sex differences. Furthermore, many factors complicate the discovery of sexual dimorphisms, such as varied methodologies for measuring fractures, biased study designs overlooking the status of sex hormones, limited transparent reports, and underpowered studies in males. The relatively small sample size used in research also limits the ability to reveal sex differences in diabetic osteoporosis.^10^

## Molecular mechanisms underlying sexual dimorphism in osteoporosis

Bone development and remodeling throughout life are complex and occur differently in women and men.^72^ To gain insights into sex differences, it is necessary first to understand bone biology and then the cellular and molecular pathways underlying sexual dimorphism in osteoporosis.

### Sex variations in bone cell functions and key signaling pathways

Approximately 80% of the skeletal mass comprises cortical bone, which provides strength and protection. The remaining 20% of the bone mass is made up of trabecular bone, which provides structural support and permits bone flexibility.^73,74^ Bone mass plays a critical role in determining the risk of osteoporosis and fragility fractures, and it exhibits obvious sex differences. In females, bone mass often peaks in the early 20 s and undergoes a rapid decline after menopause, which is closely related to estrogen levels.^75^ However, in men, bone mass frequently peaks in the late 20 s, with testosterone primarily responsible for the rapid and extensive increases in bone mass, strength, and dimensions. Typically, males have a higher peak bone mass and thicker cortical thickness than females (Fig. 1c).^76,77^

Bone tissues comprise three major types of bone cells, namely, osteocytes, osteoblasts, and osteoclasts (Fig. 1b).^78^ Among the three types of bone cells, osteocytes are the most abundant, accounting for approximately 90%–95% of cells in adult bone tissues. Osteocytes are considered terminally differentiated bone cells arising from osteoblasts.^79^ In contrast to highly abundant and long-lived osteocytes, osteoblasts comprise only approximately 6% of the cells in adult bones and have a relatively short lifespan. Osteoblasts develop from bone marrow precursor cells, namely, mesenchymal stem cells (MSCs).^80,81^ Osteoclasts are multinucleated giant cells originating from bone marrow hematopoietic progenitors, which generate resident macrophages in various tissues and monocytes in peripheral blood and are formed by the fusion of these precursor monocytes and macrophages.^82^ Osteocytes, osteoblasts, and osteoclasts are all crucial components for maintaining normal bone function, and any disruption in their homeostasis underlies the pathogenesis of osteoporosis.^83^

Bone remodeling, also known as the bone renewal process, mainly occurs during adult life and replaces old and damaged bone without changing bone shape to maintain bone quality.^84^ Physiological bone remodeling is delicately regulated by a complex system involving multiple cells.^85^ Dysfunctional remodeling results in bone fragility, such as osteoporosis.^86^ Osteoclasts are drawn to sites of microdamage to replace old bone with new bone, and osteoclasts are known as bone-resorbing cells.^87^ Upon completion of resorption by osteoclasts, osteoblasts fill the gap resorbed by depositing new bone and completing the bone remodeling cycle. For adult bone to sustain strength and mineral homeostasis, the bone resorbed by osteoclasts needs to be equivalent to that newly formed by osteoblasts. Crosstalk between osteoblasts and osteoclasts regulates bone remodeling.^88^ Based on the interplay between osteoclasts and immune cells, osteoimmunity plays a pivotal role in bone remodeling.^89^

Osteoclasts play unique and vital roles in bone development and extracellular matrix remodeling in the adult skeleton system.^90^ Excess activity of osteoclasts favors bone resorption, leading to many skeletal diseases, such as osteoporosis.^82^ Osteoclasts develop and adhere to the bone matrix, which secretes lytic enzymes and acid to degrade bones.^82,91^ The secretion of protons is necessary for acid proteases to dissolve bone minerals and digest the extracellular matrix, facilitating resorption.^92^ Carbonic anhydrase II provides the protons for extracellular acidification by H^+^-ATPase. Vacuolar H^+^-adenosine triphosphatase coupled with Cl- conductance on the membrane plays a major role in acidification of the osteoclast-bone interface.^93,94^ However, the exact molecular mechanisms for osteoclast differentiation are still unclear. Notably, the receptor activator RANK and its ligand RANKL play essential roles as mediators in activating osteoclastogenesis and promoting bone resorption. This finding sheds new light on the molecular mechanisms underlying osteoclast differentiation and formation.^95,96^

Osteoblasts are responsible for initiating new bone formation and remodeling.^97^ Osteoblasts synthesize and secrete various extracellular proteins (e.g., type I collagen, osteocalcin, and alkaline phosphatase) essential for bone matrix formation. Collagenous and noncollagenous proteins in the matrix work with osteoblasts and osteoclasts to ensure normal bone metabolism.^98^ Osteoblasts also contribute to the mineralization of bone tissues to maintain the calcium-phosphate balance in developing bone tissues.^99^ In addition to the aforementioned primary biological functions, the other biological functions of osteoblasts include but are not limited to the following: (1) Osteoblasts produce hormones, among which the first known bone-derived hormone is osteocalcin, a calcium-binding protein and the most abundant noncollagen protein. Osteocalcin consists of 49 amino acid residues, regulating systemic glucose and energy metabolism, cognition, and reproduction.^100^ In addition, osteocalcin triggers an involuntary physical reaction in response to a threat, aiding the ability to escape from danger.^101^ (2) Osteoblasts have specific receptors for vitamins and hormones, such as vitamin D (VD), estrogen, and parathyroid hormone (PTH). Therefore, they serve as target cells for these crucial hormones.^102^ (3) Osteoblasts express receptor activator of nuclear factor-kappa B (RANK) and its ligand (RANKL), an important mediator in activating osteoclasts and holding dual roles in coupling bone formation and resorption.^103^ (4) Osteoblasts also release proteins that regulate phosphate excretion from the kidneys, such as fibroblast growth factor 23 (FGF23).^104^ (5) Mature osteoblasts may transform into bone-lining cells, which are involved in governing the influx and efflux of calcium ions in bone tissues.^105^ As previously mentioned, estrogens stimulate osteoblasts via ERs, which may explain the higher incidence of osteoporosis in postmenopausal women compared to older men. In addition to estrogen receptor 1 (ESR1), several transcription factors, such as the neural-specific transcription factor Engrailed 1, the dishevelled associated activator of morphogenesis 2, and noncoding RNAs, have been identified to have pivotal roles in osteoblast differentiation.^106,107^

Osteocytes are usually embedded in the bone matrix and responsible for the mass of the bone tissues, which are also multifunctional and dynamic cells integrating hormonal and mechanical signals.^108^ Osteocytes play a crucial role in the remodeling of bone tissues throughout life, exhibiting a range of essential functions. First, osteocytes secrete some growth factors, such as insulin-like growth factor-1 (IGF-1) and fibroblast growth factor (FGF), which stimulate osteoblasts and promote bone formation following fractures.^109^ Second, osteocytes are the major responsive cells to mechanical stimulation.^110^ Third, osteocytes facilitate the exchange of ions across the bone. Fourth, osteocytes inhibit the differentiation of osteoblasts while promoting the differentiation of osteoclasts. Moreover, osteocytes express and secrete various regulatory proteins, such as sclerostin and Dickkopf 1,^111^ which act as inhibitors of the Wnt signaling pathway. These two osteocyte-derived proteins contribute to the inhibition of bone formation.

Among hormones associated with bone homeostasis, PTH, secreted by the parathyroid gland, is crucial in regulating calcium and phosphate homeostasis and bone remodeling.^112^ Bone and the kidney are the major target organs for the effects of PTH.^113^ PTH acts on all three types of bone cells. Specifically, PTH directly stimulates osteoblasts to form bone.^106^ On the one hand, PTH indirectly activates osteoclasts to resorb bone, which further enhances the release of calcium into the blood. On the other hand, PTH also induces the release of FGF23 in osteocytes.^114^ These effects consequently lead to increased bone turnover and elevated circulatory calcium. In the kidney, PTH enhances the reabsorption of calcium and promotes the expression of 25-hydroxyvitamin D-1α-hydroxylase, which increases the production of calcitriol.^115^ Calcitriol is well known for elevating serum calcium levels by enhancing calcium absorption in the intestine, release from the bone, and reabsorption in the kidney.^114^ Owing to the pivotal roles of PTH, osteoporosis frequently occurs in patients with primary hyperparathyroidism. Interestingly, recombinant PTH was reported to increase bone density, reverse skeletal abnormalities, and improve the bone microstructure of patients with hypoparathyroidism.^116^ A combination of intermittent PTH and antiresorptive agents has been widely investigated and may be an alternative option for patients previously treated with bisphosphonates.^117^ Osteoblasts and osteocytes express the type-1 PTH receptor (PTH1R) and thus are the target cells of PTH.^118^

Following PTH/PTH analog binding to its receptor PTH1R in bone cells, a complex with low-density lipoprotein receptor-related protein 6 (LRP6) is formed. Subsequently, the complex stimulates β-catenin dependent, Wingless, and Int-1 (Wnt)-ligand-independent Wnt signaling, which in turn enhances the expression of pro-anabolic genes.^119^ Together, 19 Wnt proteins exert their effects through binding to Frizzled receptors and LRPs as coreceptors, including LRP5 and LRP6.^120,121^ To date, three Wnt signaling pathways have been discovered, including one canonical Wnt pathway (i.e., the canonical Wnt/β-catenin signaling pathway) and two noncanonical Wnt pathways, including the Wnt/Ca^2+^-dependent pathway and the Wnt/planar cell polarity pathway.^122,123^ The canonical Wnt/β-catenin signaling pathway plays a critical role in osteoporosis, mainly by promoting the differentiation of mesenchymal progenitor cells into osteoblasts.^124,125^ The importance of Wnt signaling in bone health has been evidenced by multiple studies. For example, mutations of LRP5 disrupting Wnt signaling have been associated with osteoporosis-pseudoglioma syndrome, characterized by reduced calvarial thickness.^126^

In addition to PTH-mediated activation of Wnt signaling, the complex formed by the PTH/PTH analog can stimulate the formation of cAMP through adenylyl cyclase in bone cells.^127^ The downstream effector molecules in PTH1R signaling, such as IGF-1, FGF2, and bone morphogenetic proteins (BMPs), have been reported to play essential roles in osteoblast differentiation. FGF2 also regulates bone anabolism by mediating the effects of PTH by interacting with Wnt signaling.^128^ Butyrate, a bacterial metabolite derived from gut microbiota,^129^ orchestrates PTH-mediated effects in the skeletal system, predominantly stimulating bone formation and resorption by activating the Wnt signaling pathway.^130,131^

### Effects of sex chromosome abnormalities on osteoporosis

According to the widely accepted concept, sex differences arise from the inherent inequality of the sex chromosomes in XX and XY zygotes, which also cause sex disparities in osteoporosis phenotypes.^132,133^ For instance, skewed X inactivation causes variable sex-specific expression of plastin 3 (PLS3), thereby resulting in X-linked osteoporosis.^14,134^ *PLS3* polymorphisms have been linked with skeletal fragility and early-onset osteoporosis in children and young adults.^135^ Additionally, pathogenic mutations in *PLS3* strongly correlate with fractures in men and postmenopausal osteoporosis in women.^136^ Notably, considerable variations in *PLS3* have been detected in women with heterozygous X chromosomes. These variations caused by *PLS3* escaping X-inactivation or X-inactivation of the mutant allele may explain why women are less severely affected than men in X-linked osteoporosis.^137^

**Fig. 2:**
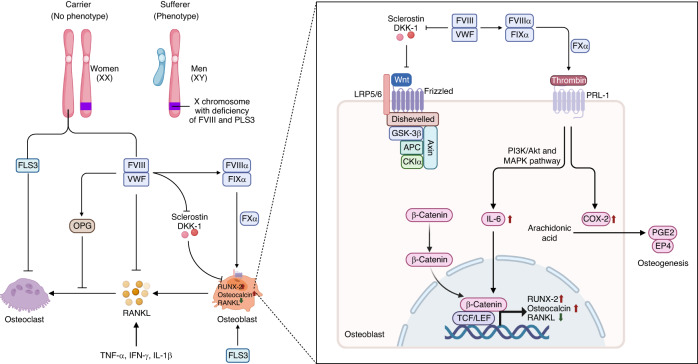
X-linked osteoporosis in males. X-linked osteoporosis is most likely more common in males, given the higher rates of mutation in genes, such as *PLS3* and factor VIII (*FVIII*), that are located on the X chromosome. *PLS3* deficiency induces an imbalance between bone resorption and formation, resulting in insufficient mineralization in osteoblasts, increased bone resorption in osteoclasts, and dysregulation of mechanosensing in osteocytes. A missing or defective clotting protein, factor VIII (FVIII), may directly disrupt bone homeostasis via the RANK/RANKL/OPG pathway in hemophilia A (HA) patients. The FVIII/VWF complex inhibits RANKL and increases the activity of OPG, thereby promoting osteogenesis. Activated FVIII detaches from VWF, binds to FIX, and then activates FX to FXa, which is responsible for the conversion of prothrombin into thrombin. Thrombin binds to PRL-1 to increase the production of IL-6, which further enhances the expression of RUNX2 and osteocalcin, decreasing the expression of RANKL. FVIII or FIX regulates the Wnt/β-catenin pathway and reduces the production of sclerostin to further inhibit the Wnt signaling pathway. OPG osteoprotegerin, RANKL receptor activator of nuclear factor-kappa B ligand, TNF-α tumor necrosis factor α, IFN-γ interferon-γ; IL-1β, interleukin-1β, IL-6 interleukin-6, MAPK mitogen-activated protein kinase, COX-2 cyclooxygenase 2, PGE2 prostaglandin E2, EP4 PGE2 receptor 4, RUNX2 runt-related transcription factor 2

Osteoporosis is associated with X-linked genetic disease, particularly in males.^138,139^ Osteoporosis and low bone mass are prevalent comorbidities in hemophilia A (HA), a congenital X-linked recessive genetic disease.^140–143^ The high prevalence of osteoporosis in HA patients has been reported to be closely associated with VD deficiency and hemophilic arthropathy.^144,145^ In HA patients, the missing or defective expression of factor VIII (FVIII) disturbs bone homeostasis via the RANK/RANKL/osteoprotegerin (OPG) axis. The FVIII-induced production of thrombin regulates bone metabolism by upregulating interleukin-6 (IL-6), runt-related transcription factor 2 (RUNX2) and osteocalcin, as well as changing the cytokine profile (Fig. 2).^146^ Global knockout of the *FVIII* gene induced trabecular bone accretion in male mice and diminished cortical compartment accretion in female mice. Furthermore, it resulted in repressed bone formation in male mice but increased resorption in female mice.^147^

Another sex chromosomal abnormality with a high risk of osteoporosis is Klinefelter syndrome (KS), a common genetic condition characterized by an extra copy of the X chromosome in males. X-linked copy number variants may play a role in the elevated risk of osteoporosis, affecting approximately 40% of individuals with KS.^148^ Patients with KS exhibit lower BMD than matched healthy people, which can be predicted by muscle mass, a history of testosterone treatment, age at diagnosis and bone markers.^149^ Notably, a cross-sectional study showed that the bone mass phenotype in patients with KS was not associated with testosterone levels or the androgen receptor (AR) CAG polymorphism.^150^ Interestingly, even men with KS who have adequate testosterone levels may have reduced bone mass, and testosterone replacement therapy does not always improve bone density in KS patients. Several factors, including low insulin-like factor 3 (INSL3), VD deficiency, reduced estrogen level, unfavorable fat/muscle ratio, and high follicle-stimulating hormone (FSH), may contribute to osteoporosis in KS patients.^151^

### Major sex hormones in bone metabolism

Similar to sex chromosome abnormalities, sex hormones, including estrogen, androgens and testosterone, also contribute to sex differences in the risk and pathophysiology of osteoporosis.^152^ Sex steroids act as essential regulatory sex hormones in bone modeling and remodeling processes by targeting bone cells, mainly osteoblasts and osteoclasts. The production of sex hormones changes dramatically and differently in men and women during their lifetime and is the most important factor for sex differences in osteoporosis (Fig. 1).

#### Estrogen’s powerful influence on bone homeostasis and its deficiency in osteoporosis

Among sex steroids that affect bone homeostasis and health, estrogen is undoubtedly the most important sex hormone in women and a major hormone in men.^17^ 17β-estradiol (estradiol, E2) is well known as the most potent estrogen with three major receptors, including classical estrogen receptor alpha (ERα) and estrogen receptor beta (ERβ), as well as nonclassical G protein-coupled estrogen receptor 1 (GPER1).^153^ Estrogen, which reaches its peak during a woman’s 20 s and declines after menopause,^75,154^ plays a critical role in tightly controlling bone mass in women. Postmenopausal bone loss, which is due to the associated decline in estrogen and usually occurs 5–15 years after menopause, is characterized mainly by an accelerated loss of trabecular bone, hence why postmenopausal osteoporosis is also referred to as trabecular osteoporosis.^155^ According to a study on perimenopausal and postmenopausal women, the annual loss of BMD was approximately 2%–5% in the first several years of menopause followed by a notable increase in BMD loss, reaching 20%–30% at the femoral neck and 30%–40% at the spine ten years following the onset of menopause.^156^

Estrogen is also associated with age-related osteoporosis in men.^75^ A deficit of estradiol in older males contributes to reduced BMD due to a high turnover rate, poor microarchitecture, and rapid bone loss.^10,157^ The direct role of estradiol deficiency in men’s osteoporosis is well supported by studies of men with congenital ER deficit (also referred to as congenital estrogen resistance) and aromatase deficiency.^158^ Men with aromatase deficiency presented a phenotype similar to that seen in estrogen-resistant patients.^159^ Nevertheless, unlike estrogen-resistant men, men with aromatase deficiency respond well to estrogen therapy and experience an increase in BMD following treatment.^160^ Inhibition of aromatase significantly reduces the urinary markers of bone resorption and formation in men at a mean age of 68.^161^ Although the clinical phenotypes of the two human models of estrogen deficiency differ, adult men with congenital aromatase deficiency benefit from high-dose estradiol treatment.^162^ These findings provide direct evidence that estrogen deficiency is essential in bone loss in older men.

**Fig. 3:**
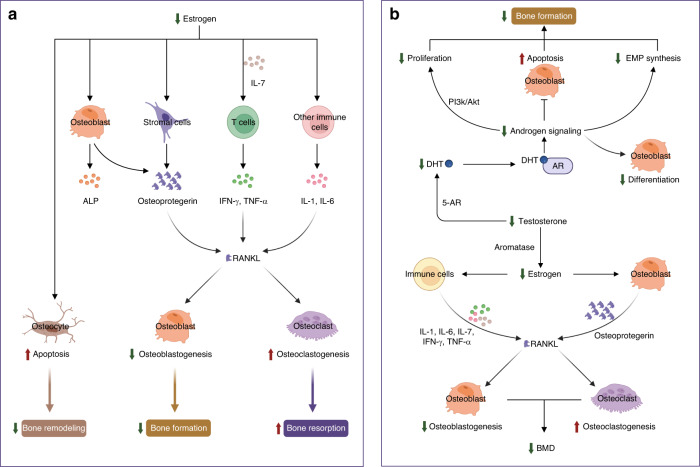
Mechanisms of sex steroid hormones on bone homeostasis. **a** Mechanism of estrogen action on bone cells. Estrogen is the most important sex hormone in women who undergo a hormonal shift in 17β-estradiol levels, transitioning from perimenopause to early postmenopause. The declining estrogen levels after menopause directly enhance the apoptosis of osteocytes to reduce bone remodeling, indirectly decrease osteoblastogenesis, and increase osteoclastogenesis by regulating RANKL. Estrogen regulates RANKL by acting on stromal cells and immune cells by changing cytokine profiles. **b** Schematic of the effects of male sex hormones on bone cells. The male sex hormone testosterone is an important regulator of bone cells, mainly osteoblasts and osteoclasts. Decreased testosterone levels in older men induce a decrease in DHT, which further represses the proliferation and differentiation of osteoblasts, increases apoptosis of osteoblasts, and reduces the synthesis of EMP. The decline in testosterone results in reduced estrogen levels, which further directly or indirectly decreases osteoblastogenesis and increases osteoclastogenesis to reduce BMD. ALP alkaline phosphatase, Bcl-2 B-cell lymphoma-2, ERα estrogen receptor alpha, ERβ estrogen receptor beta, FasL Fas ligand, IL-1 interleukin-1, IL-6 interleukin-6, IL-7 interleukin-7, IFN-γ interferon-γ, RANKL receptor activator of nuclear factor kappa B ligand, TNF-α tumor necrosis factor-alpha, Akt serine/threonine-protein kinase, AR androgen receptor, 5-AR congenital 5-alpha-reductase, BMD bone mineral density, DHT dihydrotestosterone, EMP erythromyeloid progenitor, PI3K phosphatidylinositol-3 kinase

The molecular mechanisms of estrogen in bone homeostasis are complex. Over the past few decades, numerous studies have focused on the effects of estrogen on bone cells during growth, maturation, remodeling, or turnover in adults, shedding light on how estrogen deficiency contributes to osteoporosis.^78,94,163,164^ First, estradiol inhibits inflammation by repressing key pro-osteoclastic cytokines in T cells, including tumor necrosis factor (TNF), IL-1, IL-6, and IL-7.^165^ The enhanced release of these cytokines in response to estrogen deficiency is associated with increased osteoclast differentiation and activity, as well as bone resorption (Fig. 3a).^166,167^ Estradiol also reduces inflammation by downregulating the nuclear factor-κB (NF-κB) pathway, inhibiting osteoclast formation and bone resorptive activity.^168,169^ Second, estradiol promotes apoptosis of bone-resorbing osteoclasts (Fig. 3a).^170,171^ Estradiol induces the expression of Fas ligand (FasL), a member of the TNF subfamily, in osteoblasts at the transcriptional level by binding to ERα. The activation of Fas/FasL signaling thereby induces osteoclast apoptosis in bone tissues.^172,173^ Estradiol also inhibits osteoblast-driven osteoclastogenesis by regulating the membrane association of RANKL, another member of the TNF family, to decrease the amount and activity of osteoclasts.^174^ Third, estradiol has pro-osteoblastic activity by upregulating the antiapoptotic protein B-cell lymphoma-2 (Bcl-2), leading to a net increase in new bone formation.^175,176^ 17β-estradiol relieves oxidative damage in osteoblasts by increasing the expression of miR-320-3p and decreasing RUNX2.^177^ In aging female mice, the increase in FGF23 during a mild phosphate challenge is higher than that in male mice, possibly due to the direct effect of estradiol on osteocytes.^178^ Additionally, estradiol directly upregulates the expression of alkaline phosphatase and bone-specific alkaline phosphatase, both of which are recognized as biomarkers for osteoblast differentiation, thus leading to an increase in osteoblasts and the formation of new bone (Fig. 3a).^173^

ERs can affect the biological role and signaling pathways in the sex dimorphism of bones.^179^ ERα and ERβ are abundantly expressed in osteocytes, osteoblasts, osteoclasts, and immune cells, with different but overlapping distributions in females and males.^179^ At the mRNA level, ERα is approximately 10-fold more abundant than ERβ in trabecular bone.^180^ In addition, ERα plays a major role in mediating bone responses to estrogen in both sexes, whereas ERβ has minor protective effects in females and no significant effects in males.^99^ Increasing attention has recently been directed toward the emerging roles of ERα in mechanotransduction signaling pathways in articular chondrocytes.^181^ In line with the above notion, bone formation was shown to be enhanced via upregulation of ERα in the process of fracture healing in mice following an application of mechanical strain induced by high-frequency low-magnitude vibration.^182^ Specifically, ERα activation induces the differentiation of MSCs into osteoblasts, the differentiation of osteoblasts into osteocytes, and apoptosis of osteoclasts.^1,183,184^ The effect of estrogen on cortical and trabecular bone mass is mediated by a direct effect on osteoblasts and osteoclasts via ERα.^155^ A novel resveratrol oligomer derivative exhibited potent anti-osteoporosis effects in ovariectomized rats by activating ERβ, indicating the potential effects of ERβ in osteoporosis in females.^185^ Although GPER1 is expressed in osteoblasts, osteocytes, and osteoclasts, relatively few reports exist on its role in bone homeostasis.^186^

Previous animal studies of bone cell-specific deletion of ERα have gained insights into its biological role and signaling pathways in the sex dimorphism of bones. ERα inactivation in extrahypothalamic neurons during late puberty increased bone length in female mice but not in male mice, suggesting that central ERα signaling affects bone growth and radial bone expansion via the growth hormone (GH)/IGF-1 axis, specifically in females.^187^ ERα deletion in both osteoblasts and chondrocytes induced substantial trabecular bone loss and reduced cortical bone periosteal and endosteal diameters in female mice but not in male mice, indicating that ERα in osteoblast progenitors and hypertrophic chondrocytes played sexually different roles in bone mass regulation.^188^ Deleting ERα in osteocytes decreased the trabecular bone mass only in male mice.^189^ ERα knockout was associated with decreased bone turnover and increased trabecular bone volume in both female and male mice, while ERβ deletion had the same effects only in female mice.^99^ ERα, but not ERβ, in osteocalcin-positive osteoblasts was suggested to boost the late stage of bone regeneration in female mice.^190^ Ovariectomy induces enhanced expression of microRNA-148a and osteoporosis, and microRNA-148a increases apoptosis of osteoblasts by inhibiting ERα in female mice.^191^

These data demonstrate that estrogen is a crucial sex hormone that governs bone metabolism in both women and men and protects bone tissues through a variety of molecular mechanisms. Further insights into the sex- and age-related differential effects and mechanisms of estrogen and ERs will help in the development of tailored strategies to prevent and treat osteoporosis in specific populations.

#### Androgens in bone health and osteoporosis

Testosterone acts as an essential regulatory sex hormone in bone modeling and remodeling processes by targeting osteoblasts and osteoclasts. Testosterone can be converted to DHT, the most powerful androgen, which directly binds to the AR to induce androgenic activity (Fig. 3b).^17,192^

Endogenous inadequacy of androgens due to disease may cause osteoporosis. Hypogonadism is a clinical and biochemical syndrome in which the testes cannot produce physiological concentrations of testosterone, usually indicated by a serum testosterone level of less than 300 ng/dL.^193^ Due to acquired mild hormonal deficiencies, hypogonadism has been identified as one of the most common causative factors for secondary osteoporosis in men. Men with hypogonadism presented lower BMD and poorer bone microarchitecture.^194^ Additionally, it has also been shown that testosterone benefits the BMD of hypogonadal men more than it does men with physiological levels of testosterone.^195,196^ Some previous studies have shown that testosterone replacement therapy (TRT) enhanced BMD and improved bone turnover markers and microarchitecture in men with hypogonadism.^10,197–200^ The results of testosterone trials also showed organ-specific effects for testosterone treatment in older males.^201^ Despite the increasing use of testosterone supplementation for the prevention and treatment of osteoporosis, there are no studies supporting the use of TRT in reducing the risk of fractures in men. Therefore, further studies are needed to obtain deeper insights into the pathophysiology and clinical effects of testosterone on bone to improve the use of testosterone for this indication.

Androgen deficiency due to aging or androgen deprivation therapy in patients with prostate cancer is another leading cause of osteoporosis. The decline of testosterone during aging is associated with reduced BMD, osteoporosis, and enhanced risk of fractures.^8,202^ Rapid bone loss and severe deterioration of microarchitecture occur in prostate cancer patients undergoing androgen deprivation therapy.^203^ However, the exact levels of testosterone that may cause the development of osteoporosis and the onset of osteoporotic symptoms have not been established due to conflicting results in human studies.^204^

Testosterone has numerous functions in the skeletal system. Testosterone regulates periosteal apposition and increases bone growth, making male bones larger than female bones.^205^ Testosterone protects bones by modulating nonskeletal factors such as muscle strength.^206^ Moreover, testosterone stimulates the differentiation and proliferation of osteoblasts while suppressing the maturation and resorptive activity of osteoclasts. Testosterone deficiency in ovariectomized male rats stimulated RANKL production by osteoblasts, and elevated levels of RANKL enhanced bone resorption by promoting osteoblast differentiation, leading to decreased BMD (Fig. 3b).^207–209^ Additionally, bioactive estrogen (estradiol, E2) and DHT, converted from testosterone by aromatase, contribute to the protective effects of testosterone on bone (Fig. 3b).^209–212^ Of the three types of bone cells, osteoblasts are the direct target of testosterone-derived DHT and estradiol, which promotes bone formation and remodeling by binding to AR and ERs in men.^208,213^ Notably, aromatase inhibitors result in a lower BMD and higher fracture risks in postmenopausal women with ER-positive breast cancer, suggesting a protective role of testosterone in women with estrogen deficiency.^214^ Androgen also interacts with vitamin D_3_ (VD3) to prevent the development of osteoporosis in men.^215^ Treatment with DHT was reported to increase serum VD3 in mice, which further confirmed an association between androgens and VD homeostasis.^216^ Studies by other researchers found that VD can suppress the peripheral conversion of androgen to estrogen catalyzed by aromatase.^217^ Estrogens, but not androgens, increased the gene expression of VD-binding protein (DBP), a protein primarily responsible for the transport of circulating VD.^218^ Interestingly, DBP gene polymorphisms were associated with BMD and the risk of fractures in male patients with osteoporosis.^219^ Genome-wide association analysis of circulating DBP identified two variants associated with serum DBP levels.^220^ Although the beneficial effects of VD on bone, such as in the absorption and reabsorption of calcium, have been well documented, research on the interplay of VD and androgen needs further study to better understand its role in male osteoporosis.

AR mediates the maintenance and modulation of bone homeostasis. AR-deficient mice exhibited decreased bone mass, reduced volume of trabecular and cortical bone, and osteopenia in endochondral bones due to enhanced bone turnover and resorption.^155^ Specific deletion of AR in bone cells, such as osteoblasts and osteocytes, reduced the trabecular bone mass. However, the absence of AR in cells other than osteoblasts and osteocytes can also regulate bone mass. Conditional knockout of AR in neurons of male mice exhibited obvious loss of cortical thickness and strength even with sufficient androgen.^221^ Deletion of AR in B lymphocytes increased their numbers, suggesting androgen-mediated regulation of lymphocytes.^222^ Testosterone inhibited the expression of IL-6 from immune cells, reducing the pro-osteoclastic effect.^223^ Conversely, testosterone deficiency may enhance the unfavorable impact of IL-6 on BMD. Blocking AR also significantly impaired bone repair in female rats.^223–225^

In addition to older males with low testosterone levels, osteoporosis has been observed in young men with normal testosterone levels, which is categorized as idiopathic osteoporosis.^10^ Idiopathic osteoporosis is a heterogeneous disorder in young adults with decreased osteoblast function and impaired bone acquisition, and the exact pathological mechanism remains unclear.^226^ Men with low serum IGF-1, a major GH mediator that plays a crucial role in bone remodeling, have an elevated fracture risk, especially hip and vertebral fractures.^10^ In contrast, higher serum IGF-1 levels were reported to be associated with idiopathic osteoporosis in premenopausal females.^227^ The expression of the IGF-1 receptor on circulating osteoblast progenitor cells can predict the bone formation rate and effects of teriparatide treatment in patients with premenopausal idiopathic osteoporosis.^228^ Given that abnormally low levels of IGF-1 have been closely linked to suppressed bone formation in most male patients with idiopathic osteoporosis at a young age, those patients may benefit from recombinant GH as an alternative therapy.^10,229^

### Sex hormone-mediated oxidative stress and protective autophagy in osteoporosis

**Fig. 4:**
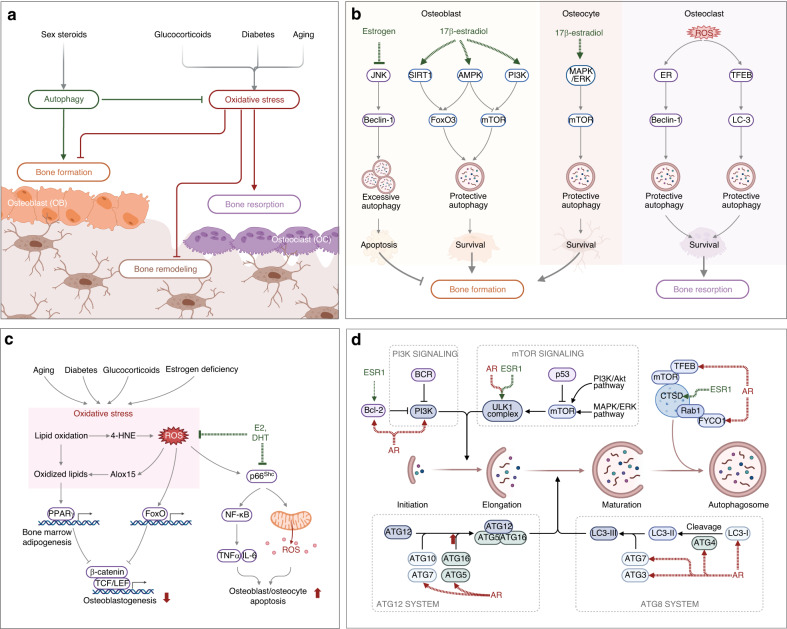
The role of sex hormone-mediated oxidative stress and protective autophagy in osteoporosis. **a** Glucocorticoids, diabetes, and aging induce reactive oxygen species (ROS) and increase oxidative stress, which is a major mechanism of osteoporosis. Excessive oxidative stress disrupts bone homeostasis by promoting bone resorption and inhibiting bone formation and remodeling. Sex steroids, especially estrogen, can promote protective autophagy to enhance bone formation. **b** 17β-estradiol promotes protective autophagy in osteoblasts and osteocytes via FOXO3 and mTOR signaling to enhance bone formation. Estrogen also inhibits excessive autophagy in osteoblasts via JNK signaling to decrease apoptosis. Excessive ROS increase protective autophagy in osteoclasts to promote survival and bone resorption. **c** Aging, diabetes, glucocorticoids, and estrogen deficiency all increase ROS to induce excessive oxidative stress to repress osteoblastogenesis through TCF/LEF signaling. Oxidized lipids in bone marrow further enhance adipogenesis by activating PPARγ signaling. Crosstalk between ROS and p66^Shc^ promotes the apoptosis of osteoblasts and osteocytes, which can be repressed by E2 and DHT. **d** Schematic illustration of autophagy from initiation, elongation, maturation, and autophagosome. Key molecules under the regulation of sex hormone receptors, AR and ESR1, are indicated by dotted lines. ROS reactive oxygen species, AR androgen receptor, ER estrogen receptor, ESR1 estrogen receptor 1, E2 17β-estradiol, DHT dihydrotestosterone, 4-HNE 4-hydroxynonenal, TCF/LEF T-cell factor/lymphoid enhancer-binding factor, JNK c-Jun n-terminal kinase, AMPK adenosine 5’-monophosphate (AMP)-activated protein kinase, MAPK mitogen-activated protein kinases, ERK extracellular regulated protein kinases, TFEB transcription factor EB, PI3k phosphoinositide 3-kinase, FOXO forkhead Box O, ULK1 unc-51-like kinase 1, PPARγ peroxisome proliferator-activated receptor γ

Age-related increases in reactive oxygen species (ROS) are inversely related to age-associated decreases in BMD and bone strength, which may contribute to age-related osteoporosis.^230^ Accordingly, the beneficial effects of estrogen and phytoestrogens on bone are attributed at least in part to their antioxidant properties (Fig. 4a).^231^ In addition to aging, some age-related metabolic diseases are also associated with increased ROS levels.^230^ For instance, hyperglycemia and insulin resistance were shown to result in increased ROS production in T2DM patients due to the promotion of mitochondrial respiration.^55,232^ Additionally, GC-induced ROS upregulate oxidative stress-related gene expression in osteoblasts and inhibit Wnt-induced osteoblastogenesis.^233^

Oxidative stress plays a critical role in osteoporosis progression.^234^ Long-term exposure to oxidative stress disrupts bone homeostasis, thereby contributing to osteoporosis.^235^ Aging-induced oxidative stress leads to enhanced osteoclast activity and reduced osteoblast activity.^236^ Aging, diabetes, GCs, and estrogen deficiency can all promote the production of ROS to induce excessive oxidative stress, which decreases osteoblastogenesis through TCF/LEF signaling.^237^ Oxidative stress also promotes the production of proinflammatory cytokines and miRNAs to increase osteoclastogenesis and decrease osteoblastogenesis.^238^ ROS are important components regulating the differentiation of osteoclasts.^239^ As a kind of ROS, H_2_O_2_ causes oxidative damage to multiple intracellular macromolecules, eventually leading to cell senescence and death.^240^ Notably, excessive oxidative stress can be neutralized and lowered by antioxidants, such as glutathione and N-acetyl cysteine (NAC). Therefore, they may induce beneficial effects on bone or even hold potential as therapeutic agents for osteoporosis.^97,234^ Sudachitin, a polymethoxyflavone from *Citrus sudachi*, suppresses inflammatory bone destruction and osteoclastogenesis by decreasing ROS production in osteoclast precursors.^241^ The powerful antioxidant NAC was shown to inhibit orchiectomy-induced osteoporosis in mice by repressing osteocyte senescence.^242^ Vitamins K1 and K2 protect osteoblasts from H_2_O_2_-induced oxidative damage, suggesting a protective role of vitamin K in the mineralization, formation and remodeling of bones.^243^ Inhibiting p66^Shc^ decreases oxidative stress and promotes osteogenesis, which can be repressed by E2 and DHT (Fig. 4c).^244,245^ Collectively, defense against oxidative stress represents a potent way to prevent osteoporosis in the elderly.^246^

Autophagy is a potential protective mechanism to protect bone cells from oxidative stress-induced damage and maintain homeostasis (Fig. 4b).^247,248^ Under oxidative stress, excessive ROS can trigger autophagy through multiple signaling pathways, such as ROS/FOXO3,^249,250^ ROS/AMPK,^251^ ROS/Akt/mTOR,^252^ HIF-1α/BNIP3,^253^ MAPK/ERK,^254^ and ROS/JNK/c-Jun.^255^ It has been well documented that autophagy, a self-degradation of damaged organelles, aggregated or misfolded proteins and other macromolecules, represents an essential key mechanism for preserving organismal and cellular homeostasis, including bone homeostasis.^256^ Thus far, three types of autophagy (macroautophagy, chaperone-mediated autophagy, and microautophagy) have been identified, of which macroautophagy is most closely related to human diseases, including osteoporosis.^257^ Dysfunctional autophagy induced by aging may be an essential mechanism for developing age-related osteoporosis.^155,258^ Autophagic activity is also involved in growth factor-mediated effects on bone. For instance, an interplay between BMPs, strong osteogenic growth factors, and autophagic activity has been identified. The ligands of BMPs participate in modulating autophagy levels in muscle-related disease by regulating energy metabolism.^259,260^ β-catenin-dependent canonical Wnt signaling, a validated signaling pathway with a critical role in the differentiation of stem cells to osteoblast lineage cells and the formation of mature osteoblasts in osteogenesis, is negatively correlated with autophagy.^261^ Autophagy is also involved in the modulation of osteoclastogenesis and osteoclast function.^262^ For instance, GCs induce bone loss by increasing autophagy in osteoclasts via the PI3K/Akt/mTOR signaling pathway.^263^ Many autophagy-related proteins (ATG), such as ATG5, ATG7, ATG4B, and MAP1LC3, have been reported to be important in promoting bone resorption.^264^ Targeting Atg7 repressed the activity of osteoclasts in ovariectomized mice while inhibiting autophagy in osteoblast aggregate bone loss in estrogen deficiency cases.^265,266^ Cell-specific deletion of Atg7 in osteoblasts reduced bone formation by triggering endoplasmic reticulum stress.^256^ Modulating autophagy is a promising strategy for treating osteoporosis. Advanced glycation end product (AGE) accumulation in hyperglycemia causes senescence of bone marrow MSCs and induces senile osteoporosis, which can be reversed by enhancing mitophagy by overexpressing sirtuin-3.^267^ Enhancing autophagy in bone mesenchymal stem cells (BMSCs) promoted bone formation and osteogenic differentiation by activating mTOR and triggering the WNT/β-catenin pathway.^268^ Specifically, enhancing autophagy in osteoblasts by degrading the Notch intracellular domain boosted the differentiation of osteoblasts and relieved osteoporosis.^269^ Estrogens, especially 17β-estradiol, promoted protective autophagy in bone cells to inhibit ROS and promote cell survival (Fig. 4b).^270,271^

Growing evidence suggests that the expression levels of many autophagy genes are regulated by sex hormones, such as estrogens and their receptors, contributing to the distinction between autophagy-mediated osteoporosis in men and women (Fig. 4d).^272–276^ For instance, ATG3 is regulated by AR only, whereas Unc-51-like autophagy activating kinase 1 (ULK1) is the target gene, with its transcription level regulated by both AR and ERα. Bioinformatics analysis has revealed that ERα potentially regulates 19 autophagy genes, and 12 autophagy genes are potential target genes of ERβ in humans, with their expression levels regulated by ERs at the transcriptional level. Further analysis of the gene-related pathways has found that these genes play functional roles in the induction of phagophores, expansion, and fusion with lysosomes in autophagy.^276^ Among these autophagy genes potentially regulated by sex hormones and their receptors, ULK1 was confirmed to play a direct role in bone homeostasis and osteolytic metastasis.^277^ By silencing or overexpressing ULK1 in vitro and in vivo, ULK1 has been demonstrated to be directly involved in modulating OC differentiation via the ULK1/docking protein 3/spleen tyrosine kinase axis, while ULK1 upregulation inhibits OC-mediated bone resorption and thereby impedes bone loss.^277^ 17β-estradiol increases the expression of sirtuin-1 to promote autophagy via the AMPK/mTOR signaling pathway and inhibits apoptosis by activating FOXO3a signaling in osteoblasts.^270^ Estrogen deficiency in female rats decreases autophagy and enhances apoptosis in osteocytes, while estrogen replacement therapy enhances the viability of osteocytes by repressing apoptosis and maintaining autophagy.^278^ As illustrated by proteomic analysis, estrogen promotes autophagy in human osteoblasts during differentiation to promote survival and mineralization by upregulating RAB3 GTPase-activating protein.^279^

Research on sex-specific autophagy genes is still in its early stages. Interestingly, some sex-specific autophagy genes are regulated by either AR or ERs, while others are regulated by both AR and ERs (dual regulation). It is still unclear whether dual regulation occurs in the same or different biological processes. It has yet to be determined whether these sex-specific autophagy genes play functional roles related to sex differences in osteoporosis. Further insights into these autophagy genes regulated by sex hormones and receptors may provide new targets for developing novel therapeutic approaches and help tailor treatment strategies for osteoporosis.

### Estrogen and testosterone in GC-induced osteoporosis

**Fig. 5:**
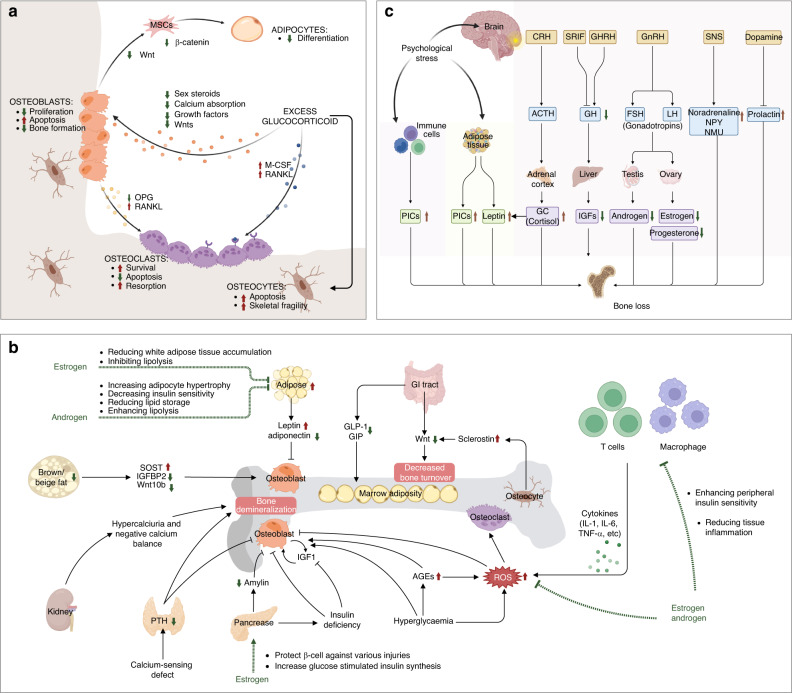
Pathological mechanisms of osteoporosis induced by diabetes, glucocorticoids, and psychological stress. **a** Glucocorticoid (GC) inhibits the proliferation and increases the apoptosis of osteoblasts by downregulating Wnt signaling, repressing the production of sex steroids, decreasing calcium absorption, and reducing growth factors. GC decreases the differentiation of adipocytes in bone marrow by decreasing the Wnt/β-catenin pathway. GCs also promote the apoptosis of osteocytes by increasing the influx of Ca^2+^. GCs decrease apoptosis and promote the survival of osteoclasts by upregulating RANKL and downregulating M-CSF. **b** T2DM-associated osteoporosis is characterized by decreased bone turnover and impaired microarchitecture, which has a complex pathological mechanism and involves multiple signaling pathways. T2DM affects bone metabolism, demineralization, bone marrow adiposity, and calcium balance through hyperglycemia. Other coexisting conditions in T2DM, such as obesity, impaired renal function, hypercalciuria, reactive oxygen species (ROS), more advanced glycation end products (AGEs) accumulation, inflammation, and peptides in the gastrointestinal (GI) tract, contribute to the higher prevalence of osteoporosis in T2DM patients. Estrogen and androgen can scavenge ROS directly or indirectly by changing cytokine profiles and exert protective effects by nonskeletal mechanisms such as the regulation of adipose tissues. Estrogen protects pancreatic β cells and increases insulin production to indirectly alleviate diabetic osteoporosis. **c** Psychological stress contributes to osteoporosis through the hypothalamic‒pituitary‒adrenal (HPA) axis and the brain-immune connection. Psychological stress promotes bone loss by regulating growth hormones, glucocorticoids, sex hormones, and pro-inflammatory cytokines. MSCs mesenchymal stem cells, M-CSF macrophage colony-stimulating factor, OPG osteoprotegerin, RANKL receptor activator of nuclear factor kappa-B ligand, AGEs advanced glycation end products, ROS reactive oxygen species, SOST sclerostin, GLP-1 glucagon-like peptide-1, GI tract gastrointestinal tract, GIP gastric inhibitory polypeptide, IGF insulin-like growth factor, GC glucocorticoid, CRH cortisol-releasing hormone, GnRH gonadotrophin-releasing hormone, GHRH growth hormone-releasing hormone, SNS sympathetic nervous system, NPY neuropeptide Y, NMU neuromedin U, ACTH adrenocorticotrophic hormone, GH growth hormone, FSH follicle-stimulating hormone, LH luteinizing hormone, PICs pro-inflammatory cytokines

Despite their high therapeutic effectiveness, the use of GCs induces various side effects, including GC-induced osteoporosis.^42,280^ Usually, bone loss may occur soon after the initiation of GC treatment, and the fracture risk may increase within months following treatment.^40^ Specifically, GCs inhibit the proliferation and differentiation of osteoblasts, elevate the apoptosis of osteoblasts and osteocytes, and increase osteoclastogenesis.^281,282^ GCs induce autophagy in osteoblasts and inhibit their proliferation by downregulating the Wnt and MAPK signaling pathways.^258^ For osteocytes, GCs induce their apoptosis by increasing the influx of Ca^2+^ and promoting the Pyk2-JNK signaling pathway.^283^ In addition, GCs promote the differentiation and maturation of osteoclasts and increase the number of osteoclasts by upregulating RANKL, thereby prolonging the osteoclast lifespan and resulting in bone loss (Fig. 5a).^39^

Crosstalk between GCs, estrogen and testosterone plays key roles in osteoporosis. GCs inhibit gonadotropin secretion, resulting in decreased production of estrogen and testosterone, as well as increased bone resorption, which consequently contributes to osteoporosis.^284^ Testosterone in hypogonadal men has been shown to prevent bone loss at the lumbar spine in GC-induced osteoporosis, while the selective estrogen-receptor modulator raloxifene decreased fractures in postmenopausal females.^285^ Regarding osteoporosis, GCs and estrogens have opposing actions on multiple signaling pathways. Both GCs and estrogens can regulate the MAPK signaling pathway, in which estrogens increase the phosphorylation of p38 MAPK. In contrast, GCs decrease the phosphorylation of ERK-MAPK, leading to different effects on the proliferation of osteoblasts.^286,287^ Furthermore, estrogens upregulate and phosphorylate the Akt protein, while GCs downregulate it.^288,289^ As mentioned earlier, estrogens and phytoestrogens protect bone by alleviating GC-induced osteoporosis and antagonizing GC side effects. When translating these scientific findings into clinical practice, sex- and age-specific differences should be considered to enhance efficacy and decrease side effects. For instance, estrogens rapidly decline in postmenopausal women, so prolonged medication is recommended when they require GC therapy to diminish the risk of osteoporosis. Male patients needing GC treatment may benefit from combining GCs with phytoestrogens, such as poncirin,^290^ quercetin,^291,292^ genistein,^293^ trigonelline,^294^ and icariin,^295^ to reduce the risk of developing GC-induced osteoporosis.^270,296^ Overall, phytoestrogens show some benefits, but their effectiveness in the context of GC-induced osteoporosis is not well established and needs further investigation.

### Diabetic osteoporosis

As a metabolic disorder, the influence of diabetes on bone tissues and cells is complex.^297,298^ T1DM patients exhibit enhanced systemic inflammation, bone loss, and fracture risks, in which IL-10 is essential in promoting osteoblast maturation.^299^ Boys with T1DM exhibited significantly lower BMD at several sites than boys without T1DM, while these obvious differences were not observed in girls with T1DM. Factors affecting BMD in boys and girls with T1DM also differ notably.^300^ Male T1DM patients showed lower 25(OH)D levels and higher PTH levels than control individuals, while no such differences were found in females with T1DM.^301^ All these reports suggest sex differences in the pathological mechanisms underlying T1DM-induced osteoporosis. Ovariectomy-induced estrogen deficiency aggregated T1DM-induced expression of TNF-α in osteoporosis in female mice, suggesting that TNF-α may play a more important role in females than in males in T1DM-induced osteoporosis.^302^ Endogenous BMP-6 was shown to be reduced in male T1DM mice, which contributed to bone loss.^303^ Most studies on T1DM-induced osteoporosis are carried out using male animal models, so data on female animals are somewhat limited.

In contrast to T1DM, T2DM is a chronic metabolic disease characterized by insulin resistance. The pancreas still produces insulin, but not at sufficient levels, and the tissues and cells cannot respond appropriately to insulin. Unlike T1DM, which usually onsets at a young age, T2DM commonly occurs at an older age and frequently coexists with obesity. The prevalence of T2DM exhibits obvious sex differences during a lifetime (Fig. 1c). Similarly, males are more vulnerable to nutritional challenges and more likely to develop insulin resistance and hyperglycemia than females in nearly all animal models.^304^ However, female T2DM patients exhibited a higher relative risk of cardiovascular diseases and mortality than males, suggesting that hyperglycemia may neutralize the beneficial effects of estrogen.^305^ T2DM-associated bone disease is characterized by decreased turnover and impaired bone microarchitecture (i.e., enhanced cortical porosity and reduced cortical volume).^66^ Although there was seemingly normal BMD or even greater levels of areal BMD in T2DM patients than in individuals without T2DM, a higher risk of developing fragility fractures was reported in T2DM patients.^67^ However, the exact underlying mechanisms need to be further elucidated. Several diabetes-associated risk factors that may contribute to fragility fractures in T2DM patients have been identified, including obesity, insulin resistance, poor glycemic control, micro- and macrovascular complications, exogenous insulin therapy, and accumulation of AGEs.^66,67^ Furthermore, T2DM and aging enhanced the production of senescent cells in various tissues, including bone tissues, in male diabetic rats.^306^ Multiple molecular pathways were activated in male diabetic rats and involved the effects of T2DM on bone metabolism, altered microarchitecture, and osteoporosis.^307^ Gene expression and glucose metabolism in osteoblasts are tightly regulated by insulin. Studies have shown that insulin stimulates the differentiation and proliferation of osteoblasts by binding to insulin receptors under normal conditions.^308,309^ Due to insulin resistance and long-term exposure to uncontrolled hyperglycemia in T2DM, the metabolism of osteoblasts is severely impaired, which directly disrupts bone homeostasis mainly through osteoblast-mediated bone formation.^310^ In addition to the aforementioned direct effects, T2DM also induces impaired renal function, hypercalciuria, aberrant AGE accumulation in collagen, inflammation, and diabetic retinopathy, which also facilitate the higher prevalence of osteoporosis in patients with diabetes (Fig. 5b).^56,308^ In rodent models of T1DM and T2DM, estrogen (17β-estradiol) has been demonstrated to display protective effects on pancreatic β cells through ERs, and β cells are involved in maintaining bone homeostasis by regulating osteocalcin.^311^ Based on these findings, it has been proposed that enhanced ER action is a promising therapeutic approach to preserve functional β cell mass in diabetic patients (Fig. 5b).^311^ Physical exercise-induced skeletal irisin relieved T2DM-induced bone loss in female rats.^312^ High insulin levels enhanced cortical bone mass and influenced microstructure by regulating osteoblast- and osteoclast-related gene expression in male KK-Ay mice.^313^ Canagliflozin improved bone microarchitecture by regulating the differentiation of osteoblasts via AMPK/RUNX2 signaling in male T2DM mice.^314^ It should be pointed out that the majority of the current studies on diabetic osteoporosis are carried out in either male or ovariectomized female animal models, which is far from support elucidating sex differences. Animal models mimicking premenopausal diabetic women should be established. If studies are simultaneously carried out on both male and female animals, comparing results and reaching conclusions on sex differences will be easier. It is now important to find clues of sexual dimorphism of diabetic osteoporosis in risk factors and biochemical markers of both sexes.

In summary, diabetes is among the leading causes of secondary osteoporosis, and the diabetic population is increasing worldwide.^315^ In China, it has been reported that the overall prevalence of total diabetes was 12.8%, ranging from 6.2% in Guizhou to as high as 19.9% in Inner Mongolia, and a higher prevalence was observed in men than in women.^291,316^ Therefore, basic research and clinical studies must be accelerated to prevent and treat diabetic osteoporosis, especially in countries with a high prevalence of diabetes and a rapid growth of aging populations.

### Psychological stress-induced osteoporosis

Both biological and psychosocial factors contribute to sex differences in the risks and outcomes of osteoporosis, among which psychosocial stress shows a greater influence on women than on men.^317^ Although mental disorders and osteoporosis have distinct pathological mechanisms, several recent studies show that mental health disorders are strongly associated with osteoporosis,^318,319^ and notably, this negative association differs between women and men.^320,321^

A nationwide longitudinal study reported that patients with posttraumatic stress disorder had a much higher risk of osteoporosis (HR: 2.66, 95% CI [1.91, 3.71]) later in life. It should be noted that 76.3% of the subjects in this study were females.^322^ The influence of psychological stress on postmenopausal osteoporosis patients with or without depression was also investigated. It was found that the lumbar vertebra and femur dual-energy X-ray absorptiometry scores were significantly lower in women with osteoporosis and depression than in control patients.^323^ In another study, the correlation between chronic psychological stress and osteoporosis was investigated in 2 327 patients with depression and 21 141 matched control individuals. The results reported a significantly lower BMD in the vertebra, distal radius, and proximal femur and higher levels of bone resorption markers in patients with depression than in control individuals without depression.^324^ Analysis of the sex-specific difference between depression and osteoporosis indicated that women with depression responded more strongly to psychological stress and that osteoporosis was approximately three times more common in women than in men.^323^ In addition, women were more vulnerable to depression-associated low bone mass, whereas men with depression displayed significantly more bone loss than women with depression.^324^ Aside from the direct effects of psychological stress, pharmacological agents for treating mental disorders such as major depression or posttraumatic stress disorder exert drug-induced side effects on bones, leading to lower BMD and a higher risk of osteoporosis and fractures in patients with mental diseases.^325^ Antipsychotics can also induce hyperprolactinemia, which further increases the risk of osteoporosis.^326^

Serum 25(OH)D concentrations are associated with anxiety levels in postmenopausal women, indicating the role of VD in psychological stress and osteoporosis.^327^ Several studies have suggested that stress hormone signaling mediated by the hypothalamic‒pituitary‒adrenal (HPA) axis and the brain-immune connection may be essential contributors. Growth hormones, GCs, and inflammatory cytokines may mediate the adverse effects of psychological stress on bone loss, leading to osteoporosis (Fig. 5c).^328,329^ How psychological stress regulates the development of osteoporosis still needs to be elucidated.

Due to the effects of sex hormones, sex chromosomes, or other intrinsic or extrinsic differences between the sexes (e.g., bioavailable estrogen, physical activity) in osteoporosis, it is necessary to take sex-specific genetic and environmental factors into serious consideration when planning future etiological studies of osteoporosis.

## Strategies and challenges in the management of osteoporosis

**Fig. 6:**
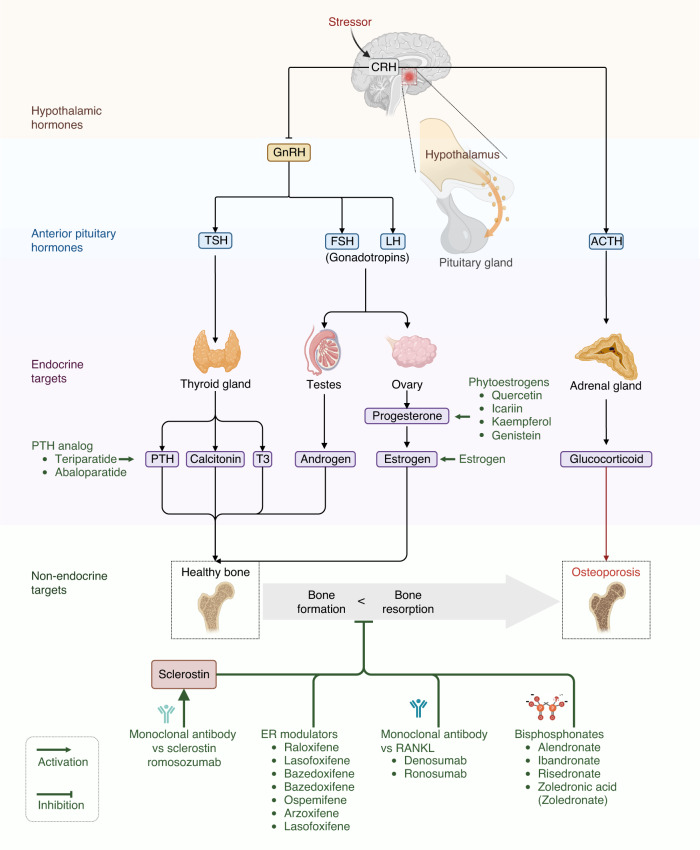
Overview of current mainstream treatments for osteoporosis. Numerous endocrine system hormones, including the hypothalamic‒pituitary‒adrenal (HPA) axis, PTH, androgen, estrogen, and glucocorticoid, all tightly regulate bone homeostasis and may be powerful targets for osteoporosis treatment. Current mainstream treatments for osteoporosis are indicated in green. CRH cortisol-releasing hormone, GnRH gonadotrophin-releasing hormone, ACTH adrenocorticotrophic hormone, TSH thyroid stimulating hormone, FSH follicle-stimulating hormone, LH luteinizing hormone, PTH parathyroid hormone, T3 triiodothyronine, ER estrogen receptor, RANKL receptor activator of nuclear factor kappa-B ligand

As a metabolic disorder, bone homeostasis of formation and resorption is under the tight control of the endocrine system, and the disruption of homeostasis results in osteoporosis. Various osteoporosis treatments have been developed to primarily strengthen bone and reduce the risk of osteoporotic fractures (Fig. 6).^330,331^ Based on their pharmacological mechanisms, the current medications for osteoporosis fall into two main categories^332,333^: (1) antiresorptive agents that exert therapeutic effects through suppressing osteoclast-mediated resorption and, in turn, reduce the rate at which bones breakdown or resorb and (2) anabolic medications that activate osteoblasts and thereby stimulate new bone formation.^334,335^ Given the sex- and age-specific differences in osteoporosis and its related complications, such as osteoporotic fracture, treatment strategies need to be tailored for women and men.^336^ The following section summarizes the current treatment options for osteoporosis for both sexes, discusses the challenges, and proposes future directions for improving the tailoring of medications.

### Current treatments for osteoporosis in female and male patients

#### Antiresorptive therapies for osteoporosis

Antiresorptive agents have been the mainstay of treatments for osteoporosis in the past three decades and have shown proven effects in decreasing fracture risks and increasing BMD. However, they have been reported to cause adverse reactions, such as atypical femoral fractures and jaw osteonecrosis, resulting in reduced reliance on these medications.^119^ To date, antiresorptive pharmaceuticals approved for osteoporosis comprise five major classes: bisphosphonates, sex hormones, selective estrogen receptor modulators, monoclonal antibodies, and calcitonin.^337^

##### Bisphosphonates

Bisphosphonates are the first-line, most widely used, and least expensive antiresorptive agents for osteoporosis.^338,339^ Bisphosphonates are usually considered the first option for osteoporosis, and approved bisphosphonates include alendronate (Fosamax),^340,341^ ibandronate (Boniva), risedronate (Actonel),^342,343^ and zoledronate or zoledronic acid (Reclast).^344–347^ These drugs have shown anti-fracture efficacy with a good safety profile and are recommended for treating osteoporosis and individuals with prior osteopenia (an increased risk of osteoporosis) or a history of fragility fracture.^348,349^ Bisphosphonates are effective at increasing BMD and decreasing fracture risk in patients with diabetic osteoporosis.^350^ As shown by a retrospective study enrolling 7 830 subjects, preadmission use of bisphosphonate was associated with superior survival among critically ill patients.^351^ The American College of Physicians recommends that male osteoporosis patients should be offered bisphosphonates to decrease the risk of vertebral fractures.^352^

In addition to the therapeutic effects of alendronate in treating male patients with primary osteoporosis, the agent is effective for males with a high risk of developing fractures.^340^ A network meta-analysis demonstrated that alendronate reduces osteoclast-mediated bone resorption and matrix breakdown, resulting in increased BMD at the lumbar spine and femoral neck, thereby decreasing the risk of new vertebral fracture.^353^ Using a national database, alendronate was found to reduce the risk of hip fractures with sustained safety in patients above 80 years old and prior fractures.^354^ Oral alendronate administered weekly also increases BMD, thereby improving GC-induced bone loss among male and female patients.^355^ In men with hypogonadism or eugonadism, alendronate significantly decreased the incidence of vertebral fracture compared with control individuals (0.8% in patients treated with alendronate vs. 7.1% in the control patients).^340^

Zoledronate is recommended for male patients needing intravenous treatment and is the only treatment demonstrated to decrease fracture risks in men.^10^ In addition to effects on fracture risk, zoledronate induced fewer vascular events, decreased the incidence of cancer, and reduced the mortality of osteogenic women older than 65 years old compared with a placebo.^356^ A retrospective cohort study using the Danish and Swedish health registries reported that zoledronate increased the risk of heart failure and death when compared to bisphosphonates. However, it is important to note that there was no increase in cardiovascular mortality in zoledronate users in comparison to those using bisphosphonates or untreated control individuals. This observation may be due to the higher baseline risks present in patients who begin zoledronate treatment.^357^

Current data about atrial fibrillation and bisphosphonates remain contradictory.^358,359^ However, due to adverse skeletal effects, the use of bisphosphonates in patients with the highest risk of future fractures dropped from 15% in 2004 to 3% in 2013 in the US.^360^ Considering that bisphosphonate use in male patients treated with sex steroids helps restore the patients’ eugonadal status, it may be worthwhile investigating whether the combined use of bisphosphonates could improve the clinical efficacy of testosterone.

##### Estrogens and phytoestrogens

Pharmacological therapy for osteoporosis began in the 1940s when estrogen was found to reverse the negative calcium balance in postmenopausal women.^160^ Estrogen facilitates the acquisition of bone mass during puberty and plays a crucial role in bone homeostasis.^17^ Estrogen levels sharply decline in menopausal women, which directly contributes to a decrease in BMD and predisposes this sex- and age-specific population to osteoporosis and a high risk of osteoporotic fractures.^154^ In animal studies, ovariectomy-induced depletion of estrogen biosynthesis caused osteoporosis, which has been used to develop an animal model of postmenopausal osteoporosis.^361,362^

The efficacy and safety of estrogens and phytoestrogens in patients with osteoporosis, especially postmenopausal female patients, have been extensively investigated.^363^ Hormonal therapy with estrogens reduced the incidence of all osteoporotic fractures in postmenopausal women in the Women’s Health Initiative (WHI) randomized controlled clinical trial. In the Women’s Health, Osteoporosis, Progestin, Estrogen (HOPE) clinical study program, 822 women were randomly assigned to three different treatment groups: the combined equine estrogen (CEE) group, the CEE plus medroxyprogesterone acetate (MPA) group, or the placebo group, and all participating women were given 600 mg/d elemental calcium. Compared to the placebo group, the hormone treatment groups showed a significant increase in BMD, specifically a 0–1.5% increase in hip BMD and a 0–3% increase in spine BMD. Furthermore, this clinical trial also observed drug-related side effects, such as breast pain and vaginal bleeding, with greater risk in the CEE + MPA group than in the placebo group.^364^ In addition to direct effects on osteoporosis, menopausal hormone therapy reduces the risk of T2DM, enhances insulin sensitivity, and improves glycemic control, which may exhibit indirect and long-term benefits to bone health.^365^

In addition to estrogen treatment for osteoporosis, naturally occurring phytoestrogens, such as quercetin,^296^ icariin,^366,367^ kaempferol,^368,369^ and genistein,^370,371^ have shown therapeutic effects in female patients with osteoporosis.^372,373^ Isoflavones, a kind of phytoestrogen with lower binding affinity than endogenous estrogens, can act as an ERβ selective ligand and an inhibitor of testosterone 5-α reductase to maintain the balance of estrogen/androgen to promote bone health.^374^

However, side effects induced by hormone treatment (e.g., breast pain and vaginal bleeding) were also observed.^364^ Overall, estrogen replacement therapy can be offered to perimenopausal or early postmenopausal women experiencing moderate to severe symptoms, as the benefits tend to outweigh the risks.^375^ However, it is important to note that long-term estrogen therapy has been associated with increased risks of cardiovascular events. Therefore, the route of administration, dose, and duration of estrogen treatment should be individualized after careful consideration.^376^ The excessive risk of breast cancer associated with estrogen replacement therapy has also raised significant concerns.^377^ Although many studies have shown the high efficacy of estrogen therapy, including improved BMD and reduced fractures, it is not used as the first-line therapy for osteoporosis and osteoporotic fractures primarily due to potential adverse events. The structural similarity to estrogen enables phytoestrogens to induce estrogenic or antiestrogenic effects by binding to ERs. There are also other health concerns related to phytoestrogen treatment, such as endocrine disruption and thyroid function disturbance.^378^

The therapeutic benefits and side effects of estrogens and phytoestrogens have also been investigated in specific male patients. The deficit of estradiol, the most potent estrogen, in older males has been shown to decrease BMD and increase bone loss.^379^ Estrogen therapy has effectively improved BMD in a specific male population with congenital estrogen resistance or congenital aromatase deficiency (congenital estrogen deficiency).^380^ These clinical studies in adult men with congenital aromatase deficiency have provided evidence in support of the benefits of hormonal therapy with estradiol on this specific male population.

In summary, estrogen therapy is usually considered for older women to relieve menopausal symptoms (i.e., hot flushes). The associated improvements in bone health should be considered when weighing the benefits and the risks of estrogen treatment. Estrogen therapy is now usually recommended for postmenopausal women at high risk of fractures who cannot take other anti-osteoporosis drugs. It is recommended that the lowest effective dose of hormone be used for the shortest effective time. In addition, hormonal therapy with estrogens may be considered the main treatment and prevention of osteoporosis in an appropriate female population. Moreover, estrogen therapy needs to be individualized in the specific population of adult men with congenital aromatase deficiency or estrogen resistance.

##### Selective estrogen receptor modulators

The past decade has witnessed the development and approval of selective estrogen receptor modulators (SREMs) to treat different estrogen-responsive conditions, including postmenopausal osteoporosis and breast cancer.^381^ SREMs are structurally diverse compounds that act either as ER agonists or antagonists by interacting with ERs in target organs. Those approved or under clinical trials that have shown beneficial effects on BMD in postmenopausal women with osteoporosis (e.g., raloxifene,^382,383^ lasofoxifene,^384,385^ bazedoxifene^386^) are ER agonists in bone.^387,388^

Among SREMs, raloxifene is the only approved agent in many countries for treating postmenopausal osteoporosis and preventing vertebral fractures in women, but it has not been approved for males.^389^ As recommended in the 2018 update of French recommendations on the management of postmenopausal osteoporosis, raloxifene should be considered for women with osteoporosis who are younger than 70 years old whose risk of nonvertebral fractures is low.^390^ In clinical trials, tamoxifen and toremifene, currently recommended for patients with advanced breast cancer, have shown beneficial effects on BMD in older women after menopause. A clinical trial including 8 556 women aged 59–80 years showed that lasofoxifene at a daily dose of 0.5 mg decreased the risk of nonvertebral and vertebral fractures.^384^

Furthermore, the clinical use of SERMs has been expanded to male patients undergoing androgen deprivation therapy but needs to be carefully prescribed due to adverse events such as gastrointestinal, cardiovascular, and psychiatric effects.^391^ Two SERMs, clomiphene citrate and tamoxifen, have been used off-label in males with central hypogonadism, which enhances serum testosterone levels.^392^ Moreover, SERMs in combination with immunotherapy were suggested to benefit patients with prostate cancer by regulating the tumor immune microenvironment.^393^

Various studies have indicated the adverse effects of the long-term use of SREMs.^394,395^ SREMs have been reported to be associated with uterine cancer, venous thromboembolism, and fatal strokes, which remain a major concern for long-term therapy for osteoporosis.^396^

Currently, several new SREMs, such as bazedoxifene,^386,397^ ospemifene,^398,399^ and arzoxifene,^400,401^ either alone or in combination, are under investigation for the prevention and treatment of osteoporosis.^381,402^ As shown by a meta-analysis of PubMed, EMBASE, Web of Science, EBSCO, and Cochrane Library databases, bazedoxifene seems to have good safety and efficacy in postmenopausal women.^386^ The findings are promising, as these newly developed SREMs have potentially higher efficacy and potency than previous agents. In preclinical studies, these drugs have demonstrated their effectiveness matching that of conventional hormone replacement therapy in experimental osteoporosis in animal models with improved safety profiles.^403,404^ Human studies, including phase III clinical trials, are ongoing.

##### Calcitonin

Calcitonin is a peptide hormone released by the thyroid that binds to osteoclasts, potently suppresses osteoclast activity, and inhibits bone resorption.^405,406^ The discovery of calcitonin functions in osteoclasts and osteoporosis led to the development of synthetic human calcitonin, such as salmon calcitonin (an analog of human calcitonin), as a therapeutic drug approved for the prevention and treatment of osteoporosis.^407,408^ Calcitonin is not recommended as the first-line therapy to treat osteoporosis but is particularly appropriate for patients with high-turnover osteoporosis.^409,410^ This is mainly because other approved antiresorptive therapies, such as bisphosphonates, are more effective for treating osteoporosis and preventing osteoporotic fractures.^411,412^ The other main reason is related to calcitonin-associated side effects.^413^ For instance, the long-term use of calcitonin may elevate the risk of prostate cancer in men. In postmenopausal women, previous clinical trials of nasal calcitonin have noted rhinitis and epistaxis as the most common adverse effects, with incidence rates of 12% and 4%, respectively.^414^ Put simply, calcitonin is a relatively less effective antiresorptive therapy for osteoporosis and related fractures, and its benefit does not outweigh the risk of treating osteoporosis.^415^

##### Monoclonal antibodies

Understanding the roles of RANKL^416^ and sclerostin^417^ in bone metabolism and osteoporosis has contributed to the development of two monoclonal antibodies for treating osteoporosis and preventing fractures, namely, denosumab and romosozumab.^418,419^

Denosumab is a human monoclonal IgG2 antibody against RANKL and has been approved for the treatment of osteoporosis in postmenopausal women, women with breast cancer taking aromatase inhibitors, and men at high risk of fracture, such as prostate cancer patients undergoing androgen deprivation therapy.^420–422^ In addition, denosumab has been approved for treating GC-induced osteoporosis in both women and men at high risk of fracture. This approval was based on important findings from clinical studies on patients of both sexes who were treated with GCs and at increased risk of osteoporotic fractures.^203,423^ Both male and female osteoporosis patients were given denosumab, and notably, during the two years of treatment, the incidence of vertebral fractures, including new and worsening fractures, decreased by nearly 66%.^420^ Denosumab has also been approved for male patients with osteoporosis.^424^ Denosumab can also increase BMD in males with primary osteoporosis or prostate cancer undergoing androgen deprivation therapy.^203,425^ Despite the efficacy in improving BMD and reducing the incidence of fractures, several side effects may restrict the use of denosumab in specific patient populations. Upon discontinuation, the effects of denosumab rapidly reversed, which resulted in increased bone turnover and BMD loss,^426^ with the vertebral fracture rate increasing, even back to the level in untreated patients.^119,427^ Denosumab thus should not be stopped without alternative treatment since a sudden arrest may result in a rebound in vertebral fracture risk.^428^ Denosumab is usually considered an alternative to bisphosphonates. Without a powerful bisphosphonate at denosumab discontinuation, the incidence of vertebral fractures is high, which raises great concerns.^429^

Romosozumab is a humanized monoclonal antibody that binds specifically to sclerostin, an antagonist of the Wnt signaling pathway, resulting in the inhibition of bone resorption and stimulation of bone formation.^342,430^ Romosozumab is currently indicated for postmenopausal osteoporosis patients at high risk of fractures or osteoporosis patients for whom other treatments have failed or were intolerable.^431,432^ Romosozumab also decreased the risk of vertebral fracture in postmenopausal women at 12 and 24 months after transitioning from denosumab, and it was given for 12 months at a dose of 60 mg, which was administered subcutaneously every 6 months.^433^ A phase 2, multicenter, randomized, placebo-controlled clinical trial showed that the discontinuation of romosozumab induced large declines in BMD, with the highest decrease in the lumbar spine (9.3%).^419^ Although recent clinical studies have reported cardiovascular adverse reactions associated with romosozumab, the available evidence does not definitively establish a direct link between romosozumab and cardiovascular events.^434^ The discontinuation of denosumab is primarily linked to rapid bone loss in the majority of patients and an increased risk of vertebral fractures, underscoring the need for a careful assessment of the indications to start denosumab.^427,435,436^ Additionally, relative to denosumab, the efficacy and safety of romosozumab still needs to be evaluated for women and men at high risk of osteoporosis and osteoporotic fractures other than postmenopausal osteoporosis, such as women with breast cancer treated with aromatase inhibitors or prostate cancer patients undergoing androgen deprivation therapy.^433,437^

#### Anabolic medications for osteoporosis

Based on the hypothesis that osteocytes and osteoblasts can be irregularly stimulated to form new bones without decreasing bone resorption, anabolic therapies for osteoporosis are progressing rapidly.^119^ Anabolic medications, which act mainly by activating osteoblasts and osteocytes to promote new bone formation, are leading a new clinical paradigm.^438,439^ Based upon the findings that PTH/PTH analogs show anabolic effects on bone cells, two anabolic agents, teriparatide and abaloparatide, have been approved by the FDA for the treatment of osteoporosis.^440,441^ Teriparatide is a recombinant protein comprising the first 34 amino acids of PTH and functions as an agonist of PTH1R.^442^ Abaloparatide, a 34-amino acid peptide, shares 76% homology with parathyroid hormone–related protein (PTHrP) and 41% homology with PTH (1–34). It is a second-generation anabolic drug and acts as a selective agonist of PTH1R.^443^ Downstream effectors in PTH1R signaling are also potential targets for osteoporosis.^444,445^ For instance, targeting the canonical Wnt/β-catenin signaling pathway and its bone-specific inhibitor sclerostin has been one of the major strategies in developing therapeutic agents for osteoporosis.^446^ The recently approved monoclonal antibody romosozumab, which targets sclerostin, was designed to act on the canonical Wnt/β-catenin signaling pathway.^447,448^ However, blocking sclerostin results in the activation of Wnt signaling and possible adverse cardiovascular outcomes.^449^

Teriparatide is a recombinant form of human PTH (rhPTH) with an anabolic capacity to improve osteoblast activity and subsequently new bone formation.^343,450,451^ Teriparatide promoted bone healing in medication-induced osteonecrosis of the jaw compared to a placebo in an RCT.^452^ Among anabolic medications for osteoporosis, teriparatide is the first approved drug in this category for osteoporosis in men and women at a very high risk of fractures.^453^ A PTHrP analog (abaloparatide) is a synthetic 34-amino acid peptide analog of PTHrP with a functional role in bone formation similar to PTH in stimulating new bone formation.^454^ A post hoc analysis of the Abaloparatide Comparator Trial In Vertebral Endpoints (ACTIVE), a phase 3, double-blind, randomized, placebo- and active-controlled trial, reported that fracture events were fewer following abaloparatide treatment in patients with T2DM, and differences were not significant between groups except nonvertebral fractures in the abaloparatide versus placebo groups (*P* = 0.04).^455^ Abaloparatide was approved by the FDA in 2017 for postmenopausal women at high risk of fracture.^440^ Both teriparatide and abaloparatide exert their effects by directly binding to PTH1R, leading to stronger stimulation of bone formation rather than resorption with a net gain in BMD.^456,457^ Clinical trials in postmenopausal osteoporosis patients with a very high risk of fractures have shown that subcutaneous injection with teriparatide or abaloparatide for up to 21 months reduced incidental fractures (both nonvertebral and vertebral).^458^ Considering that treatment with either teriparatide or abaloparatide may increase serum uric acid, there should be some caution when prescribing these anabolic medications for patients with current or previous acute gouty arthritis.^459^ Teriparatide and abaloparatide may cause hypercalcemia and hypercalciuria; therefore, preexisting hypercalcemia, hypercalciuria, and a history of kidney stones are considered contraindications for the use of these drugs.^460,461^

Downstream effectors in PTH1R signaling are also potential targets for osteoporosis.^444,445^ For instance, targeting the canonical Wnt/β-catenin signaling pathway and its bone-specific inhibitor sclerostin has been one of the major strategies in developing therapeutic agents for osteoporosis.^446^ The recently approved romosozumab was designed to target the canonical Wnt/β-catenin signaling pathway, and the drug acts as a monoclonal antibody against sclerostin.^447,448^

Taken together, anabolic medications, such as teriparatide, abaloparatide, and romosozumab, have shown greater therapeutic effects in randomized clinical trials on reducing clinical fracture risks in postmenopausal women with osteoporosis and at very high risk of fracture in comparison with patients treated with oral bisphosphonates, the first-line, most commonly used, less costly antiresorptive medications for osteoporosis.^10,119,438,462^ The advantage of anabolic mediations over antiresorptive medications (e.g., bisphosphonates) in strengthening BMD and decreasing fracture risk is likely attributed to the stronger stimulation of bone formation than resorption, resulting in a net gain in BMD, an improvement in bone strength, and a reduction in osteoporotic fracture risk. Therefore, anabolic medications may be considered for osteoporosis of various causative factors (e.g., menopause in women, the use of GCs in men and women) in drug-naive male or female patients who are evaluated to have a very high risk of fractures. The use of anabolic medications may also be considered for osteoporosis patients who do not respond well to antiresorptive medications or have experienced treatment failure.^463,464^

Despite their apparent advantage in reducing the risk of fractures, particularly in postmenopausal osteoporosis in women, anabolic medications have not become the first-line pharmacologic treatment for most osteoporosis patients. This is mainly attributed to several limitations, including relatively high cost, subcutaneous administration requirements, and concerns over long-term safety.^465^ However, researchers still have great hope for anabolic agents, and initiating the remodeling cascade by activating PTH1R by teriparatide and abaloparatide will inevitably enhance resorption. Thus, there is an urgent need to understand the direct effects of anabolic agents on each cell type and the influence on bone resorption, which will help to determine the population for whom it is most suitable and optimize the treatment strategy for osteoporosis.^119^

### Challenges and future directions in the treatment of osteoporosis

Although the currently available osteoporosis medications have shown clinical efficacy in improving BMD and diminishing osteoporotic fractures (Table 1), there are undoubtedly challenges in treating different types of osteoporosis with common and distinct risk factors, including sex- and age-specific osteoporosis.^160,466^ More exquisitely designed clinical studies are needed to assess the feasibility of these treatment options in various populations.

**Table 1:** Differences in drug indications and benefits of osteoporosis medications between women and men

Medications	Sex differences in drug indications	Sex differences in benefits		
		BMD	VFs	NVFs
Alendronate	Postmenopausal women with osteoporosis Women with osteoporosis due to steroid use	✔	✔	✔
	Men with primary osteoporosis, hypogonadal osteoporosis or GIOP	✔	✔	✖
Ibandronate	Prevention and treatment of postmenopausal osteoporosis in women	✔	✔	✔
	Not approved for the treatment of osteoporosis in men	✔	✖	✖
Risedronate	Postmenopausal osteoporosis or GIOP in women Men with osteoporosis	✔	✔	✔
		✔	✖	✖
Pamidronate	Women with osteoporosis at high fracture risk	✔	✔	✔
		✖	✖	✖
Zoledronic acid	Postmenopausal osteoporosis or GIOP in women Men with primary osteoporosis Men with GIOP	✔	✔	✔
		✔	✔	✔
		✔	✖	✖
Denosumab	Menopausal women at high fracture risk Men with primary osteoporosis Men with ADT-associated osteoporosis	✔	✔	✔
		✔	✖	✖
		✔	✔	✖
Strontium ranelate	Women with osteoporosis at high fracture risk Adult men with osteoporosis	✔	✔	✔
		✔	✖	✖
Teriparatide	Menopausal women with osteoporosis Men with primary osteoporosis	✔	✔	✔
		✔	✔	✖

✓ evidence available, ✖ lack of evidence or insufficient evidence, high fracture risk is defined as having multiple risk factors for fracture or a history of osteoporotic fracture

*ADT* androgen deprivation therapy, *BMD* bone mineral density, *GIOP* glucocorticoid-induced osteoporosis, *NVFs* nonvertebral fractures, *VFs* vertebral fractures

#### Intervention threshold for initiating osteoporosis treatment in women and men

According to a recent real-world study in China, a 10-year probability of major osteoporotic fractures (greater than 7%) based on the Fracture Risk Assessment (FRAX) tool was identified as the intervention threshold for cost-effective osteoporosis treatment with zoledronate for postmenopausal women.^467^ In this respect, a certain age or a given risk factor does not represent a particular risk threshold. Therefore, further studies are needed to determine the normal ranges and thresholds in specific populations that need osteoporosis treatment, especially for postmenopausal women and older men, as well as younger women and men with multiple risk factors. A broader strategy for osteoporosis and generic bisphosphonates may be the only medication satisfying the necessary efficacy, safety, and cost.^468^ Clinical evaluation trials in males should also be performed.

#### Combined/sequential therapy

Combining anabolic agents and antiresorptive medications in sequential therapy for osteoporosis may result in greater clinical efficacy.^469,470^ The sequence in which drugs are given in combined/sequential therapy is important for the overall efficacy.^119,462^ Treatment sequence matters when sequential therapy is carried out with anabolic medications, such as PTH, teriparatide, and antiresorptive agents.^471–473^ For instance, adding alendronate to the teriparatide treatment regimen was not shown to benefit male patients with osteoporosis.^474^ Therefore, in sequential therapy for sex-specific populations, including postmenopausal osteoporosis in women and GC-induced osteoporosis in men and women who are at a very high risk of fractures, anabolic medications are recommended to be used first for a short time to improve BMD and bone strength by boosting bone formation, followed by the long-term use of antiresorptive drugs.^436,475^ Although sequential therapy reduces the risk of osteoporotic fracture with greater clinical efficacy than that of each drug alone, there are concerns about the side effects and high cost.

#### Nondrug practice in combination with current osteoporosis medications

The therapeutic benefit of osteoporosis medication in combination with alternative nondrug protocols has been increasingly recognized.^476^ The nondrug practice may include but is not limited to regular exercise, nutrition (intake of calcium and bioactive VD3), stopping excessive alcohol consumption, and quitting smoking.^477–479^ Regular physical exercise, especially weight-bearing physical activities, can maintain or increase muscle function and strengthen bones, thereby reducing fractures in both men and women.^480,481^ Regarding nutrition and supplements, it was suggested to consume foods enriched in calcium and VD or supplements of calcium and bioactive VD3 to combat osteoporosis.^482^ If dietary calcium is inadequate, supplementation is recommended. The National Osteoporosis Society Vitamin D Guideline recommends a 25(OH)D level of at least 50 nmol/L and a maintenance dose of 800 IU/d.^424^ A meta-analysis identified increased risks of stroke with intakes of calcium above 1 000 mg daily, but only in female patients.^483^ Moreover, supplementation with a polyunsaturated fatty acid-enriched diet in growing and developing mice and rats can exert positive effects on osteoblasts and decrease the risk of osteoporosis.^97^ Balanced nutrition, including protein, minerals, fruit and vegetables, should be emphasized for preventing fractures.^484^ A recent review revealed that nutrients from daily diets, such as unsaturated fatty acids, proteins, minerals, peptides, phytoestrogens, and prebiotics, can regulate bone metabolism and reverse bone loss and may prove to be an effective strategy to prevent and treat osteoporosis without subsequent side effects.^485^

#### Hormonal therapy with estrogens/phytoestrogens/androgens

When planning future clinical studies for treating osteoporosis and fractures with hormonal therapy, sex- and age-specific differences should be considered. Various doses, durations, regimens, and administration routes should also be investigated in future clinical studies to diminish side effects, especially in women with postmenopausal osteoporosis. As levels of estrogens decline sharply in women after menopause, the benefits of estrogens on bone will be diminished or might even be lost in a proportion of postmenopausal women. If GC therapy is needed for inflammatory diseases in postmenopausal women, we propose that the dosage and duration of GCs should be reduced to avoid the risk of osteoporosis. Male patients taking GCs may benefit from combination with phytoestrogens, such as quercetin, poncirin, kaempferol, genistein, and icariin, to decrease the risk of GC-induced osteoporosis.^270,292,294^ The Endocrine Society recommends testosterone replacement to males with symptoms of hypogonadism without contraindication to its use.^486^ However, the effectiveness of testosterone in increasing BMD is less obvious than anti-osteoporosis medication. Therefore, more evidence is needed.

#### More clinical trials with larger sample sizes in men

Clinical trials of anti-osteoporosis medications are more common in women than in men. Additionally, clinical trials in women usually have a larger sample size than those in men. BMD is usually used, rather than fractures, as the outcome in clinical trials in men. More clinical trials with large sample sizes in men are needed, considering the use of fractures as the outcome.^424^ In addition, the comorbidities in men are usually different from those of women, which should also be considered in designing clinical trials.

#### Raising awareness about osteoporosis and fractures, especially in men

Over the last decade, major international initiatives have been used to develop a systematic approach to decrease the risk of fragility fractures in both women and men.^487^ Considering the rapid growth of the aging population, the burden of osteoporosis is growing rapidly. However, most patients with osteoporosis and a high risk of fractures have not received any preventive care targeting future fractures.^488^ This undertreatment is usually referred to as the treatment gap, which may be due to limited awareness and poor compliance. For example, fewer male patients in the USA were tested or treated after a fracture than female patients (6% vs. 12%).^489^ Furthermore, there is a sex disparity in fracture patients and a lack of awareness of osteoporosis, particularly in older men.

## Screening approaches for osteoporosis and osteoporotic fractures

Before proper treatment, patients at risk must be identified by screening tests and assessing their risks of osteoporosis and osteoporotic fractures. Several tools have been developed, but their performance in age- and sex-specific populations varies.^10,490,491^ Screening intervals, frequencies, and potential adverse effects for sex-specific populations still need to be clarified and validated.

### Screening tests for osteoporosis and osteoporotic fractures

Studies have shown that bone measurement tests can predict osteoporotic fractures,^492,493^ especially in postmenopausal women and older men, of which dual-energy central X-ray absorptiometry (DXA) at the hip and lumbar spine is well accepted as the most commonly used test for BMD.^494,495^ The major international guidelines for the management and treatment of osteoporosis recommend using BMD as measured by central DXA to define osteoporosis and the threshold at which to initiate treating osteoporosis with therapeutic drugs to prevent osteoporotic fractures.^496,497^ Central DXA is recommended as the primary diagnostic modality for screening osteoporosis in women >65 and men >70 years of age.^498^ Moreover, central DXA is used to evaluate patients’ eligibility to be enrolled in clinical trials of anti-osteoporosis drugs.^499^

Aside from central DXA, peripheral DXA measuring the BMD of the lower forearm and heel and quantitative ultrasound (QUS) detecting bone mineral status in some peripheral sites are used as alternative tools to screen for osteoporosis and predict osteoporotic fracture risks.^500^ Although peripheral DXA and QUS are similar in predicting osteoporotic fracture risk, QUS can avoid the risk of radiation exposure.^501,502^ Compared with central DXA measurements, both peripheral DXA and QUS are performed using portable devices, making these two measurement tests easier and less costly.

### Risk assessment for osteoporosis and related fractures

Clinical risk factors for both sexes usually include a family history of hip fracture in first-degree relatives (e.g., parents), excessive alcohol consumption, cigarette smoking, and low body weight.^503^ However, the clinical risk factors for osteoporosis and osteoporotic fractures vary in specific populations, including but not limited to sex- and age-specific populations (Tables 2 and 3). In addition to bone measurement tests, central DXA, peripheral DXA and QUS may help to identify the risk of osteoporosis and osteoporotic fractures for both sexes.^504,505^

**Table 2:** Differences in osteoporosis screening recommendations between women and men

Societies/Organizations	Recommendations for Women	Recommendations for Men
World Health Organization	>65 years	No recommendation
International Society for Clinical Densitometry Endocrine Society	>65 years; OR Postmenopausal women with risk factors	>70 years; OR 50–69 years with risk factors
The U.S Preventive Services Task Force	≥65 years without previous fractures or secondary causes of osteoporosis; OR <65 years with a 10-year fracture risk ≥ that of a 65-year-old woman without risk factors	No recommendation
American Association of Clinical Endocrinologists	>65 years	No recommendation
China Association of Gerontology and Geriatrics	>65 years	>70 years
UK National Osteoporosis Guideline Group	DXA scans for women at high risk of osteoporosis	DXA scans for men at high risk of osteoporosis
Canadian Osteoporosis Society	>65 years	>65 years

*DXA* dual-energy X-ray absorptiometry, *UK* United Kingdom, *U.S.* United States

**Table 3:** Sexual dimorphism in major risk factors and biochemical markers for risk assessment and stratification in screening for osteoporosis

Risk factors/Biochemical markers	Women	Men
Unchangeable risk factors		
Older age, family history of osteoporosis, family history of fractures, previous fracture, small bone frame size, white or Asian ethnicity	✔	✔
Alterations in major sex hormone levels		
A decline in estrogen at menopause Reduction in testosterone levels with aging	✔ ✖	✖ ✔
Lifestyle-related risk factors		
Sedentary lifestyle or lack of physical activity, excessive alcohol consumption, cigarette smoking	✔	✔
Dietary risk factors		
Inadequate calcium intake, Insufficient nutrients	✔	✔
Long-term use of some medications		
Glucocorticoids Medications to reduce estrogen levels for breast cancer treatment Androgen deprivation therapy for the treatment of prostate cancer Thyroid hormone medication for an underactive thyroid	✔ ✔ ✖ ✔	✔ ✖ ✔ ✔
Underlying medical conditions and diseases		
Diabetes mellitus, hypogonadism, thyroid disorders, hyperparathyroidism, inflammatory diseases, chronic kidney disease, chronic liver disease, cancer, eating disorders, celiac disease, VD deficiency, psychological stress	✔	✔
Serum biochemical markers for risk assessment of osteoporosis		
Serum osteocalcin, BALP, PINP for bone formation, CTX-I and NTX- I for bone resorption	✔	✔
Urinary biochemical markers for risk assessment of osteoporotic fractures		
The urinary ratio of native (alpha) to isomerized (beta) CTX for risk of fractures, including hip, vertebral, and nonvertebral fracture	✔	✖

✓ evidence available, ✖ insufficient or lack of evidence

*BALP* bone-specific alkaline phosphatase, *CTX-I* carboxyterminal cross-linked telopeptide of type I collagen, *NTX-I* amino-terminal cross-linked telopeptide of type I collagen, *PINP* N-terminal propeptide of type I procollagen

A study enrolling perimenopausal Japanese women demonstrated that body weight, BMI, height, and handgrip strength were positively correlated with BMD, which can predict future osteoporosis.^506^ Menopausal status should be considered a predominant risk factor for osteoporosis and osteoporotic fractures in women. Before screening with bone measurement tests for postmenopausal women aged 65 years, clinical risk factors associated with a high risk of osteoporotic fractures should be assessed. Given that postmenopausal women can benefit from treatment in many clinical studies, an appropriate clinical risk assessment tool should determine who should undergo screening with bone measurement tests for postmenopausal women < 65 years old and with at least one risk factor.

Several clinical risk assessment tools for osteoporosis and osteoporotic fractures have been developed,^507,508^ including the Osteoporosis Index of Risk (OSIRIS),^509,510^ the Simple Calculated Osteoporosis Risk Estimation (SCORE; Merck),^508,511^ the Osteoporosis Self-Assessment Tool (OST),^512,513^ and the Osteoporosis Risk Assessment Instrument (ORAI).^514,515^ In general, these clinical risk assessment tools are moderately accurate.

There are only a few clinical trials directly evaluating the effectiveness of osteoporosis screening, particularly the differences between sexes. The most commonly used clinical risk assessment tool in predicting fractures is the FRAX (University of Sheffield, UK).^516^ The FRAX can assess a person’s 10-year risk of fracture.^517^ FRAX effectively assesses the risk for female patients, but its accuracy may be lower in males.^10^ The OST was initially developed based on data from postmenopausal women in eight Asian countries, and this screening algorithm appeared to perform well in assessing osteoporosis risk in women.^307,518^ The performance of the OST in men was also determined and compared with that of women. In a US Study, an OST score with a cutoff threshold of <1 could identify postmenopausal women with osteoporosis at the femoral neck with a sensitivity and specificity of 89% and 41%, respectively, while an OST score with a cutoff threshold of 3 can identify men with osteoporosis at the femoral neck, total hip, or lumbar spine with a sensitivity and specificity of 88% and 55%, respectively.^490^ Similarly, the performance of most screening tools for the risk of osteoporosis has been reported to vary by sex, age, and ethnicity. A systematic review reported that the performance of none of the tools is consistently better than others, and simple tools such as the OST and ORAI often performed as well or better than complex tools such as the SCORE and FRAX.^491^

### Challenges and future directions for osteoporosis screening

Various studies support screening for osteoporosis with bone measurement tests (central and DXA, peripheral DXA, and QUS) in postmenopausal women aged < 65 years at high risk of osteoporosis.^505,519^ Patients with cancer were also recommended to undergo fracture risk assessment since they have unique risk factors for osteoporosis.^151^ However, it is still inconclusive whether the benefits of screening for osteoporosis to prevent osteoporotic fractures are superior to the potential hazards caused by bone measurement tests in men.^10^

Considering the complexity of osteoporosis, particularly sex-specific differences, as highlighted in this review, we propose screening concepts for osteoporosis to prevent fractures. (1) More efforts should be made to narrow the treatment gap. Fracture prediction tools such as FRAX and imaging modalities such as DXA provide great potential to identify individuals at high risk.^520^ However, there are still many at-risk individuals missing assessment and treatment. Novel assessment methods and reduced treatment side effects will help. (2) Sex- and age-specific screening intervals and frequencies should be considered. Some studies have suggested that screening intervals are determined mainly according to baseline BMD and age. Other studies found that repeating bone measurement tests 4–8 years following the initial screening was not advantageous. Regarding frequency, the current results are still limited, especially in men. (3) There are specific risk factors for different types of osteoporosis and sex-specific differences. In addition to common risk factors for osteoporosis, specific risk factors and their predictive value should be investigated in the future in different types of osteoporosis, such as GC-induced osteoporosis and diabetic osteoporosis. (4) There are potential adverse effects of screening using bone measurement tests. Until now, no studies have examined the possible adverse effects of screening for osteoporosis in men. It has been proposed that the dangers of screening may be similar between men and women. Further efforts to elucidate this mechanism should be made. (5) Noninvasive genetic tests and biomarkers for osteoporosis and osteoporotic fractures. Osteoporosis is well documented as a bone metabolic disease with a strong genetic component.^521^ Genetic factors can explain approximately 50%–80% of the interindividual variation in BMD and 50%–70% of osteoporotic fractures.^522^ Despite the strong genetic influence and heritability, genetic tests and molecular biomarkers specific to osteoporosis and its related fractures are lacking. Thus far, no such tests and markers have been used to screen for osteoporosis. In this context, and with the sex differences in osteoporosis, future studies are needed to develop noninvasive genetic tests and biomarkers for osteoporosis and fractures in both women and men.

## Conclusions and perspectives

The sex of the patient and changes in sex hormone levels over a lifetime play pivotal roles in health and disease. Over the past decades, substantial preclinical research and clinical studies have been conducted in male animal models and men chiefly due to concerns about the influence of the hormonal cycle (e.g., menstrual cycle) on outcomes as well as the classification of women as ‘protected subjects’ in clinical trials.^14,15^ Essentially, conclusions based on one sex have been used to develop disease treatment and management guidelines for both sexes. It is important to include both the sex of the patient and hormonal changes to improve clinical outcomes.^16^ With advances in precision medicine, sex differences in disease have gained increasing attention. In line with this, two scientific organizations, the Organization for the Study of Sex Differences (OSSD) and the International Society of Gender Medicine (ISGM), have been established to facilitate in-depth research and enhance the development of sex-specific therapies. Research on sexual dimorphism in health and disease is evolving to form a new scientific discipline.

Osteoporosis and related fragility fractures commonly affect both women and men, causing a severe and growing threat to global health. Relative to the predominantly higher prevalence of primary osteoporosis in women, men are more likely to be disabled and even die from osteoporotic fractures. The misconception that osteoporosis affects only women must be addressed. Moreover, implementing care for men at high risk of fractures is highly recommended to ensure timely identification and treatment and prevent devastating consequences. In addition to the epidemiological differences between sexes, basic research and clinical studies have shed light on multiple mechanisms underlying sexual dimorphism in osteoporosis, which will help develop more effective and sex-specific osteoporosis therapies and screening tools. Given the association between diabetes, GCs, and psychological stress with osteoporosis, both basic research and clinical trials need to be promoted and prioritized in the future.

In terms of basic research, it is necessary to overcome difficulties in obtaining samples with improved state-of-the-art biobanks and carry out multiomics research usually using difficult-to-be-obtained bone samples.^523^ The impact of cell heterogeneity should also be emphasized. Single-cell RNA sequencing provides valuable information on novel cell subtypes, cell-fate transitions, cell‒cell interactions and more. Thus, these findings contribute to a deep understanding of cell heterogeneity.^524^ The combination of single-cell sequencing technology and multiomics may provide new insights for elucidating the biological mechanism of sexual dimorphism in osteoporosis. It is too time-consuming and labor-intensive to conduct separate functional studies on individual gene variants, and the results are difficult to compare and analyze. Employing high-throughput technology and artificial intelligence to detect thousands of suspected susceptibility loci may shed light on new functional evidence. Deeply elucidating sexual dimorphism in the field of metabolism, especially the mechanism of amino acid, lipid, and glucose metabolism, will provide new viewpoints for understanding the molecular mechanism of osteoporosis and finding new pharmacological targets. Animal models should be carefully designed, encompassing species differences in sex hormones and receptors. Estrogen loss should be considered in animal models when investigating aging-related disorders in women, as female mice preserve functional levels of estrogen even in old age.^525^ If gonadectomy is used, whether the surgery is performed before or after sexual maturity will significantly influence efficiency. The soybean content in the food also influences the results. If isolated tissues or cells are used, the sex and level of sex hormones in the animal and the concentration of steroid hormone analogs in the culture medium should also be considered. Since most studies have been carried out using either male or female mice, the roles and mechanisms of some important molecules cannot be compared between the sexes. It is strongly suggested to carry out animal studies in both sexes, which will facilitate the comparison and discovery of sexual dimorphism.

In clinical trials, data should be analyzed and reported based on the status of steroid hormones. Current clinical trials of anti-osteoporosis medications in men usually have small sample sizes, and the primary endpoint of most trials is BMD rather than fractures. Larger clinical trials with fractures as the primary endpoint should be carried out for male osteoporosis patients. The rapidly developing field of artificial intelligence may help to develop age- and sex-specific screening and risk assessment tools.

In terms of patient education, although great progress has been made in the treatment of osteoporosis, the treatment gap of patients with high fracture risk is increasing, especially in men. It is very important to strive to enhance compliance. The treatment gap is due not only to the gap in awareness but also to the need for further basic and medical research, development of new medicines, clinical trials, and practice. The ever-widening gap calls for urgent attention, ranging from raising public awareness, especially in older men, to motivating researchers, clinicians, and national policies to improve care for individuals at high risk of osteoporosis and associated fragility fractures. As a disease with strong sexual dimorphism, elucidating the underlying mechanisms will help to tailor therapeutic and screening strategies and to close the treatment gap.
