# Author Correction: Insights and implications of sexual dimorphism in osteoporosis

**DOI:** 10.1038/s41413-024-00329-5

**Published:** 2024-04-15

**Authors:** Yuan-Yuan Zhang, Na Xie, Xiao-Dong Sun, Edouard C. Nice, Yih-Cherng Liou, Canhua Huang, Huili Zhu, Zhisen Shen

**Affiliations:** 1 Key Laboratory of Drug-Targeting and Drug Delivery System of the Education Ministry and Sichuan Province, Sichuan Research Center for Drug Precision Industrial Technology, West China School of Pharmacy, Sichuan University, Chengdu, 610041 China; 2 West China School of Basic Medical Sciences & Forensic Medicine, Sichuan University, Chengdu, 610041 China; 3 Department of Biochemistry and Molecular Biology, Monash University, Clayton, VIC 3800 Australia; 4 Department of Biological Sciences, Faculty of Science, National University of Singapore, Singapore, 117543 Singapore; 5 Department of Biotherapy, Cancer Center and State Key Laboratory of Biotherapy, West China Hospital, and West China School of Basic Medical Sciences & Forensic Medicine, Sichuan University, Chengdu, 610041 China; 6 Department of Reproductive Medicine, Key Laboratory of Birth Defects and Related Diseases of Women and Children of Ministry of Education, West China Second University Hospital of Sichuan University, Chengdu, China; 7 Department of Otorhinolaryngology and Head and Neck Surgery, The Affiliated Lihuili Hospital, Ningbo University, 315040 Ningbo, Zhejiang China

**Keywords:** Osteoporosis, Osteoporosis

Correction to: *Bone Research* 10.1038/s41413-023-00306-4, published online 18 February 2024

Following publication of the original article,^1^ the authors reported an error in figure 1. During re-reading our previously published article entitled “Insights and implications of sexual dimorphism in osteoporosis”^1^ in Bone Research, we regrettably found that the terms of “hematopoietic precursors” and “mesenchymal precursors” in Figure 1b were wrongly reversed, which have been corrected below. Although those modifications do not affect the results and conclusion in the article, all the authors agree to correct this negligence by providing corrected Figure 1, to guarantee the accuracy of the article. The license of the corrected Figure 1 is also enclosed since it was created through BioRender. We sincerely regret and apologize for any inconvenience caused.

The original figure 1 should read:
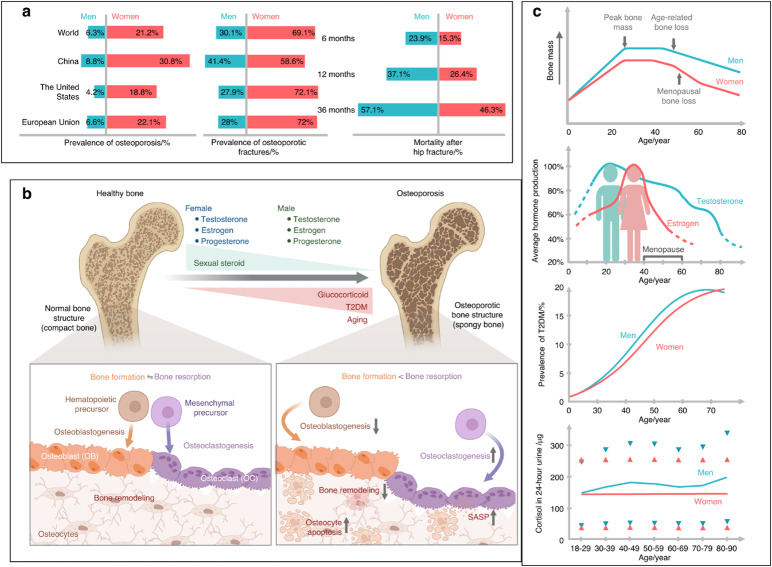


The correct figure 1 should read:
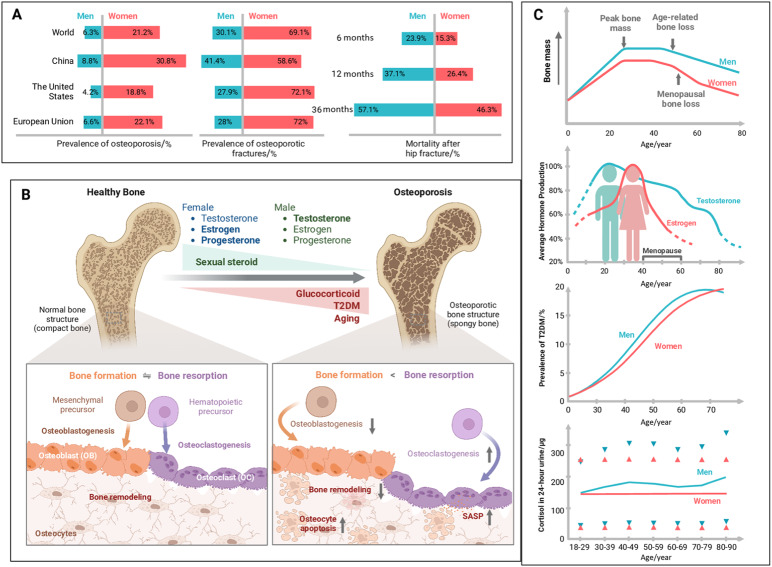


The original article [1] has been updated.
